# Open Chrono-Morph Viewer: visualize big bioimage time series containing heterogeneous volumes

**DOI:** 10.1093/bioinformatics/btae761

**Published:** 2025-01-15

**Authors:** Andre C Faubert, Shang Wang

**Affiliations:** Department of Biomedical Engineering, Stevens Institute of Technology, Hoboken, NJ 07030, United States; Department of Biomedical Engineering, Stevens Institute of Technology, Hoboken, NJ 07030, United States

## Abstract

**Summary:**

Time-lapse 3D imaging is fundamental for studying biological processes but requires software able to handle terabytes of voxel data. Although many multidimensional viewing applications exist, they mostly lack support for heterogeneous voxel counts, datatypes, and modalities in a single timeline. Open Chrono-Morph Viewer provides a straightforward graphical user interface to quickly investigate multi-timescale datasets represented as separate volume files in the common NRRD format for compatibility between toolchains. It features dynamic clipping surfaces for rapid investigation of 3D morphology and a scriptable animation API for quantitative, repeatable, publication-quality visualization. It is implemented in pure Python using common libraries to facilitate community-driven development.

**Availability and implementation:**

OCMV is available at https://github.com/ShangWangLab/OpenChronoMorphViewer for Windows, Linux, and macOS. Supporting tutorials, documentation, and installation instructions can be found in the supplementary information. Our modified Fiji I/O plugin for up to 5D NRRD file conversion is available at https://github.com/afaubert/IO.

## 1 Introduction

Advancements in live bioimaging have enabled studies of biological processes in 3D at a variety of timescales (Stower and Srinivas 2021, van Ineveld *et al.* 2022), requiring software to handle terabytes of data. For example, a study of live cardiogenesis can generate thousands of volumes across the heartbeat and stages of development, each of which comprises millions to billions of voxels. Such time-lapse 3D imaging is revolutionizing biology by associating dynamics with structure (Lippincott-Schwartz 2011), while also creating challenges in data handling for visualization and analysis (Eliceiri *et al.* 2012).

To address these high-dimensional image visualization needs, numerous open-source platforms exist, such as napari (Perkel 2021), Vaa3D (Peng *et al.* 2014), Fiji (Schindelin *et al.* 2012), and Icy (de Chaumont *et al.* 2012). However, the challenges are reflected not just by the larger size and higher dimensionality but also by the increasingly mixed imaging scales and modalities, especially when imaging significant growth of an organ or organism. Returning to the previous example, imaging cardiogenesis requires varying length scales and voxel counts over multiple time scales to accommodate the growing heart. For directly handling these heterogeneous datasets, few strategies or tools are available (Haase *et al.* 2022). As such, the approach to visualizing a time series of volumes bearing different voxel numbers and scales is limited to resampling onto a common grid, which is cumbersome and even impractical for large and high-dimensional datasets.

Open Chrono-Morph Viewer (OCMV) seeks to address this deficit by providing a simple, easy-to-learn user interface for loading a selection of independent volume files into a multiaxial timeline and using dynamically controlled clipping surfaces with real-time volume rendering to explore the morphology contained within these volumes, while maintaining data in a common, reuseable file format which remains compatible with the broad suite of existing software.

## 2 Overview

**Figure 1.**
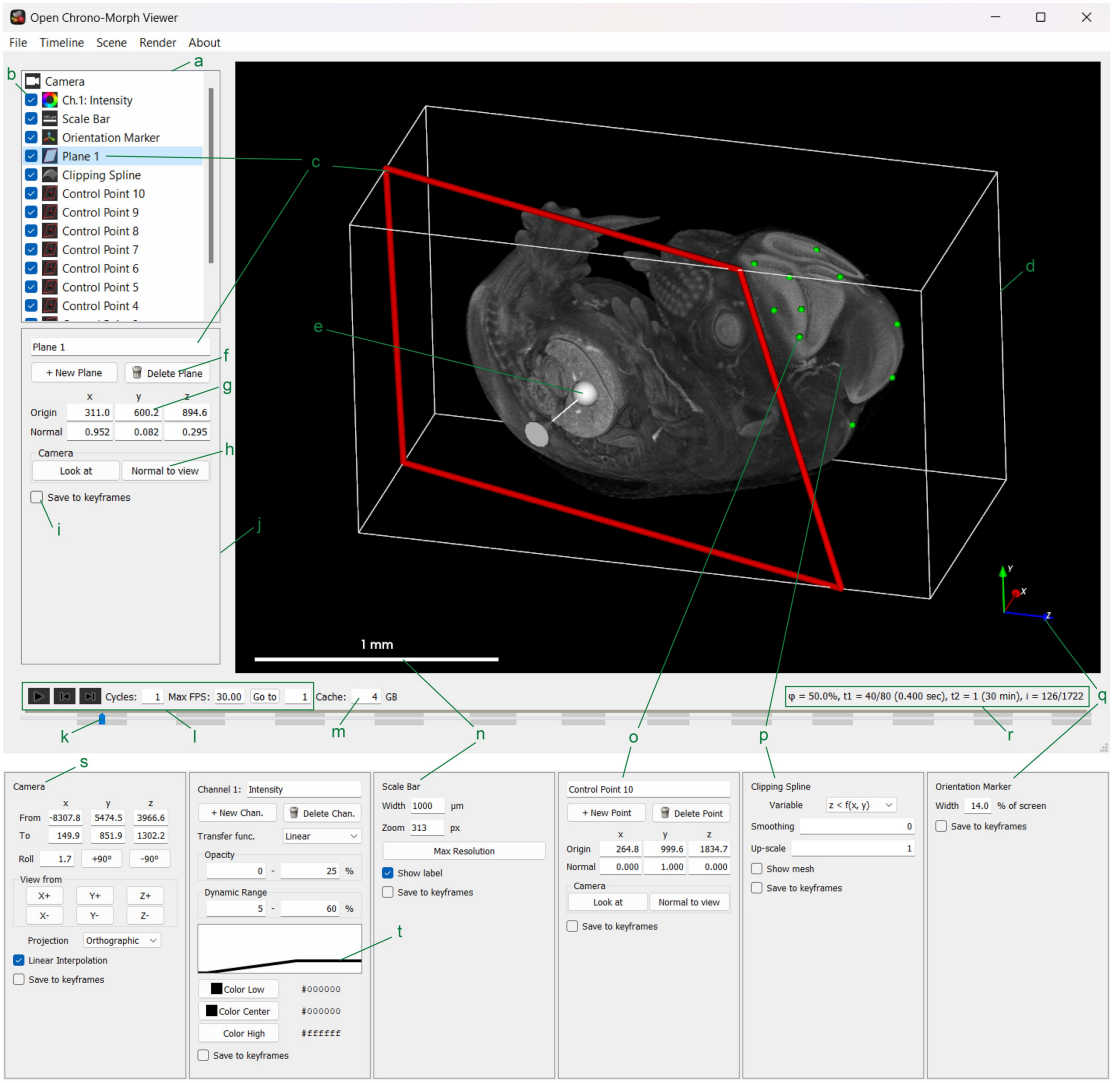
(Top) Primary window of the GUI. (Bottom) Scene item interfaces not selected in the primary window. (a) Scene list containing scene items. (b) Scene item check box. (c) Clipping plane is the active scene item. (d) Volume bounding box. (e) Plane controller. (f) Buttons to create (left) or delete (right) a plane. (g) Input boxes for the origin and normal vectors of the plane. (h) Buttons to point the camera at the plane (left) or angle the plane normal to the camera (right). (i) Checkbox indicating whether to include this scene items in saved keyframes for animation. (j) Scene panel containing the parameters of the active scene item. (k) Timeline slider selecting which volume to display. (l) Timeline controls. (m) Cache limit input box. (n) Scale bar and its scene panel. (o) A control points and its scene panel. (p) Clipping spline and its scene panel. (q) Orientation marker and its scene panel. (r), Volume index and time information label. (s) The camera’s scene panel. (t) Transfer function of the channel mapping voxel value (abscissa) to opacity (ordinate) and color (linear between saturation points). The micro computed tomography data of the mouse embryo is from the International Mouse Phenotyping Consortium (MGI:1915091) under CC-BY 4.0 license.

OCMV is written in pure Python for cross-platform support (Supplementary Note S1), delegating volumetric ray-cast rendering to the Visualization Toolkit (VTK) to achieve high performance. It features two interfaces: a Qt-based Graphical User Interface (GUI) for exploring data, and an Application Programming Interface (API) for scripting animations using the scenes and settings exported from the GUI (Fig. 1 and Supplementary Note S2).

Rather than representing multidimensional data as a single strictly uniform high-dimensional array, OCMV considers each multichannel volume to be a distinct item. This representation allows arbitrary compositions of variously typed, sized, scaled, and oriented volumes to populate the timeline and for multi-terabyte timeseries datasets to be logically split and distributed across the filesystem. This allowance of heterogeneous data allows mixed imaging modalities and data from multiple scales to be incorporated into a single investigation. The order of volumes in the timeline is automatically inferred from the plaintext tags stored in each volume header.

The rendering pipeline imposes a divide between the voxel data and the scene, i.e. the set of viewing parameters and functions that are passed to VTK and used to project the 3D voxels to the pixels of a 2D image (Supplementary Fig. S1). This allows viewing conditions to be shared between different volumes and saved at various instances to create animations between keyframes via the API (Supplementary Video S1). The scene elements include functions such as colorization of image channels (Fig. 1t), the scale bar (Fig. 1n), the camera perspective (Fig. 1s), and the planar clippers which the user can control in real-time to cut away part of the volume and see inside (Fig. 1e). The real-time clipping spline function allows the user to expose a smooth, nonplanar cutaway view which intersects a selection of control points (Fig. 1p).

## 3 NRRD and volume heterogeneity

We chose the NRRD data format due to its simplicity, flexibility, and compatibility with existing software. TIFF is also supported, but NRRD allows for the greatest functionality. An NRRD file consists of raw image data paired with a plain-text header containing the metadata (Supplementary Note S3). OCMV currently supports data types of 8- and 16-bit integers and up to four data channels for a given volume (Supplementary Fig. S2). To facilitate use of the NRRD format, we developed *tiff2nrrd*, a basic, yet efficient file conversion program for compiling TIFF images into NRRD volumes. This program is included in each installation of OCMV. In addition, we created a modified version of the Fiji I/O plugin for data conversion back and forth between up to five-dimensional NRRD and the vast collection of image formats which Fiji already supports (Supplementary Note S4).

OCMV handles each volume file in a time series independently, circumventing the limitations on the shape of volumes between timepoints, i.e. while a volume must be regularly sampled along the three spatial axes plus channel (if any), the number of samples within a group of volumes may vary arbitrarily. Likewise, the sample spacing within a group and the timestamp associated with each volume may vary. This allows volumes to be acquired at different voxel numbers, voxel scales, channel numbers per voxel, and acquisition periods. The varying spatial offset specified in the NRRD header files (Supplementary Note S3) allows volumes to be registered to one another for visual continuity during real-time playback. As an example, Supplementary Table S1 shows an OCMV-compatible dataset with 25 groups where each group contains different numbers of volumes and the volumes from each group contain different numbers of voxels. OCMV allows these volumes of different sizes and different time indices to be rendered as they are, without needing to resample the dataset onto a common grid. Furthermore, the lack of a requisite correspondence between volumes in a timeline allows data from multiple imaging modalities to be synchronized into a single timeline. For example, confocal microscopy could be used to image the earlier, small-scale embryonic development, then optical coherence tomography for later, larger-scale development, and micro computed tomography to capture development in the final stages.

## 4 Handling large datasets

The raw image format of NRRD enables not only straightforward data conversion but also disk streaming for rapid playback, while the discrete-volume-file approach allows OCMV to work with time series larger than the available RAM. In addition, OCMV reduces latency by dynamically preloading a user-defined RAM cache when possible (Supplementary Note S5).

To demonstrate OCMV’s performance when handling large sequence of volumes, we compare with Imaris (version 9.6.0, Oxford Instruments), a major commercial software widely used in bioimaging for volumetric time series (Supplementary Fig. S3, Supplementary Table S2, and Supplementary Note S6). The data conversion time (TIFF to NRRD) for OCMV is linear with respect to the raw file size, with 2000 GB data taking ∼30 h, while for Imaris File Converter (version 9.6.0), the data conversion time (TIFF to Imaris File Format) appears quadratic, with 100 GB data taking ∼80 h, and larger sizes of data (e.g. 500 GB and beyond) cannot be converted within a reasonable period (e.g. a few days). With the NRRD and Imaris files, the rendering time, i.e. the sum of time for opening the file and displaying the entire sequence once, is comparable between OCMV and Imaris for the raw file sizes ≤100 GB. Specifically, while Imaris renders faster than OCMV when operating from an SSD, the opposite is true when operating from an HDD. In tests up to 2000 GB of raw file size, OCMV maintains its linear relationship between the raw file size and the rendering time, i.e. its volumetric playback rate is independent of the number of volumes in the timeline.

## 5 Navigating two timelines

Multiple time axes address the needs arising from fast, dynamic imaging with longitudinal experiments and are especially important for studying dynamic organs repeatedly over a long period. Taking cardiogenesis as an example (Supplementary Video S2), this enables various ways to view, examine, and analyze the beating embryonic heart (fast timescale) over development (slow timescale). OCMV splices two timescales into a single slider for ease of navigation (Supplementary Fig. S4). Timing information for each volume is specified through the following metadata fields (example in Supplementary Fig. S5): the fast-varying time index within a volume group (*time index*) out of “n” possible time indices (*n times*), the physical time spacing between time indices (*period*) with units (*period unit*), and the slow-varying time index between volume groups (*group index*), along with a timestamp (*timestamp*) for indicating absolute acquisition time. Upon selecting a set of volume files, OCMV sorts the timeline first by *group index*, then by *time index*. These indices are displayed in the volume timing information label as *t*_2_ and *t*_1_, respectively, in the OCMV user interface (Fig. 1r).

## 6 Scripting reproducible animations

OCMV emphasizes quantitative controls for visualization and animation to improve reproducibility of animations and to facilitate incremental improvements. All OCMV’s functions can be controlled numerically in the GUI and selectively saved to scene files as keyframes (Fig. 1i). Thus, a particular view is re-attainable completely or partially as needed (Supplementary Video S1). Furthermore, the scene files are in JSON format which is human-readable and directly editable (Supplementary Fig. S6).

Saved keyframes can be used for animations via the API (Supplementary Note S7). OCMV includes an animation library which users can import into their Python scripts to dictate the rendering process. Users specify videos as a sequence of animation frames, where each frame contains a reference to a volumetric image, a “scene” describing the viewing conditions, and an optional list of text or image annotations to overlay upon the frame after rendering. OCMV produces the initial frame sequence by mapping the user-specified volumes and playback rate onto the output video framerate. The user then loads keyframes and applies them to relevant subsets of the frames. OCMV provides customizable interpolations for all scene items, so the user can produce intermediate scenes by interpolating specific parameters of the keyframes over time. Finally, the user adds sequences of frames to the renderer, which produces the desired images. Optionally, OCMV can compile the images into a video by calling FFmpeg through the command line. The generated animation can be easily reproduced and precisely modified (Supplementary Video S3 and Supplementary Note S8).

## 7 Conclusion

Of the freely licensed software compared (Supplementary Note S9 and Supplementary Table S3), none support multiple simultaneous clipping planes in their 3D view, only Icy and napari (with scripting) fully support multi-file time series larger than RAM, and only napari supports more than one timeline or allows for nontrivial animations, although use of these capabilities requires knowledge of Python.

While many 3D viewers are capable of handling volumetric time series, none are readily available to handle heterogeneous volumes across multiple timescales, which is increasingly required by modern bioimaging, especially in developmental biology. OCMV addresses this unmet need while maintaining simplicity in the interface and broad compatibility with existing tools. The use of Python not only provides automatic cross-platform support but also leads to straightforward integration with modern tools such as napari, the major Python-based platform for handling high-dimensional images.

## Supplementary Material

btae761_Supplementary_Data

## Data Availability

A test dataset for OCMV is publicly available on Zenodo (https://doi.org/10.5281/zenodo.13712866).
