# Supplementary material for: Open Chrono-Morph Viewer: visualize big bioimage time series containing heterogeneous volumes
Source: Bioinformatics. 2025 Jan 15;41(1):btae761. doi: 10.1093/bioinformatics/btae761 (PMC11751631; doi:10.1093/bioinformatics/btae761)
Supplement: btae761_Supplementary_Data [file btae761_supplementary_data.zip › 42097_SupplementaryInformation_Bioinformatics_OCMV_FinalFile.pdf]

Supplementary Information

Open Chrono-Morph Viewer: visualize big bioimage time series containing heterogeneous volumes

Andre C. Faubert and Shang Wang*

Department of Biomedical Engineering, Stevens Institute of Technology, Hoboken, New Jersey, 07030, USA

*shang.wang@stevens.edu

Supplementary Fig. S1: Control Flowchart ... 2
Supplementary Fig. S2: Support for four channels and 16-bit time series ... 3
Supplementary Fig. S3: Performance on large sequences of volumes ... 4
Supplementary Fig. S4: Navigating two timescales in one timeline ... 5
Supplementary Fig. S5: Example of an NRRD header file ... 6
Supplementary Fig. S6: Text-based (JSON) scene files corresponding to the GUI ... 7
Supplementary Table 1: Heterogeneous volumes in one timeline ... 8
Supplementary Table 2: Performance on large sequences of volumes, full data ... 9
Supplementary Table 3: Comparison with existing volumetric time series software ... 11
Descriptions of Supplementary Videos ... 13
Supplementary Note 1: Installation instructions ... 14
Supplementary Note 2: Overview of the graphical user interface ... 21
Supplementary Note 3: The NRRD file format and its benefits ... 26
Supplementary Note 4: NRRD file conversion ... 32
Supplementary Note 5: Adaptive RAM cache management ... 36
Supplementary Note 6: Performance on large sequences of volumes ... 37
Supplementary Note 7: Overview of the animation API ... 38
Supplementary Note 8: Reproducing and editing animations ... 40
Supplementary Note 9: Comparison with existing volumetric time series software ... 48

Supplementary Fig. S1: Control Flowchart

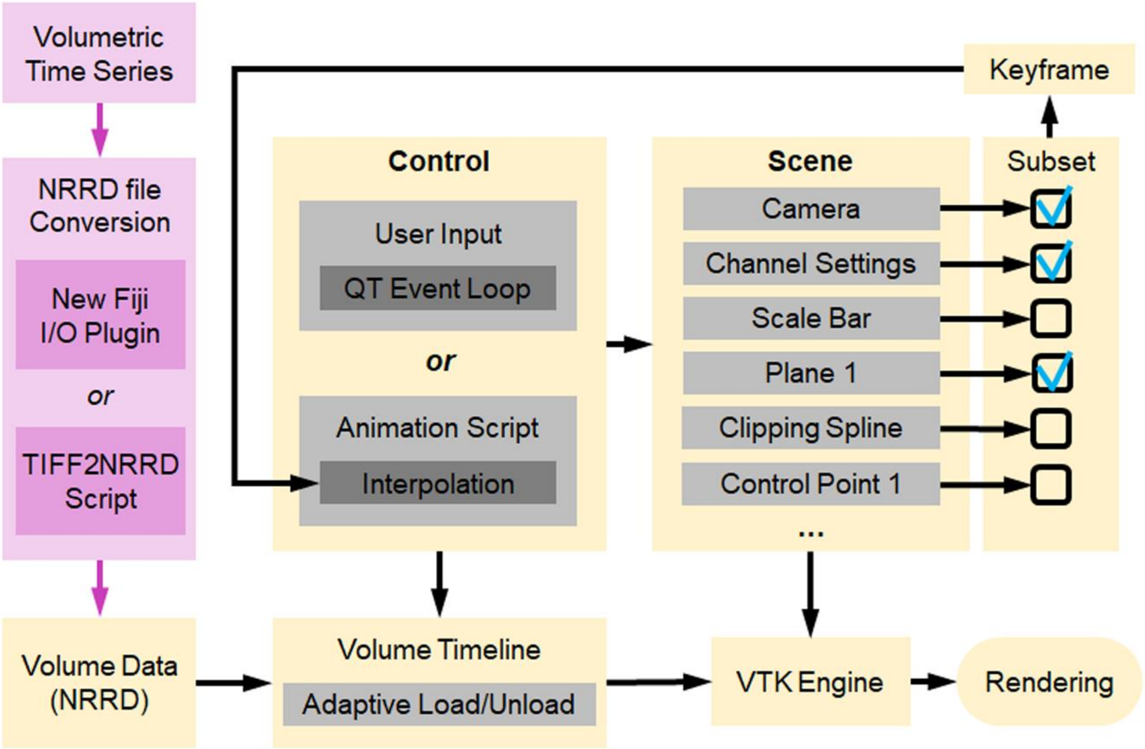

(Yellow) In *User Input* mode (via the GUI), the user manipulates the scene and volume timeline which are integrated by the VTK rendering engine to produce a rendered image. Keyframes are a subset of the available scene elements at a point in time. They can be defined in interactive mode and used with a Python script in *Animation Script* mode (via the API) to control the scene and volumes to render a sequence of video frames. **(Pink)** Through the Fiji-compatible I/O plugin or the efficient *tiff2nrrd* script (packaged with OCMV), most image data formats can be converted into NRRD to use with OCMV.

Supplementary Fig. S2: Support for four channels and 16-bit time series

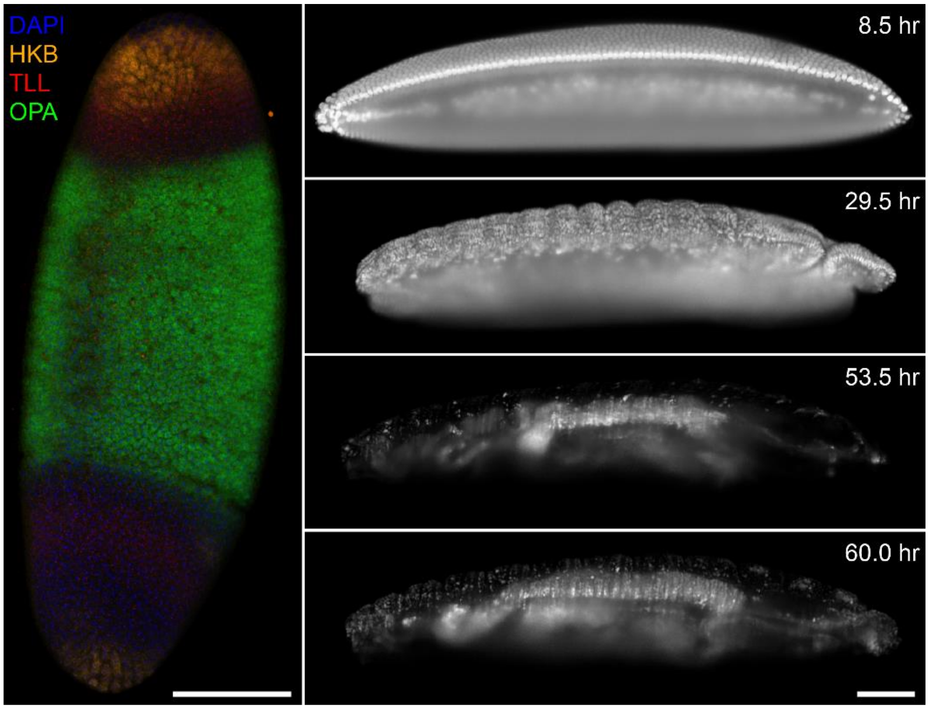

(Left) Four-channel 3D image of *Drosophila melanogaster* embryo taken by fluorescence confocal microscopy (12-bit integer). Data from BioImage Archive (Accession S-BIAD582) under CC0 license. **(Right)** Time-lapse 3D images of *Ceratitis capitata* embryonic development acquired with fluorescence light-sheet microscopy (16-bit integer). Data from Zenodo (https://doi.org/10.5281/zenodo.6448019) under CC BY 4.0 license. Scale bars: 80 µm.

Supplementary Fig. S3: Performance on large sequences of volumes

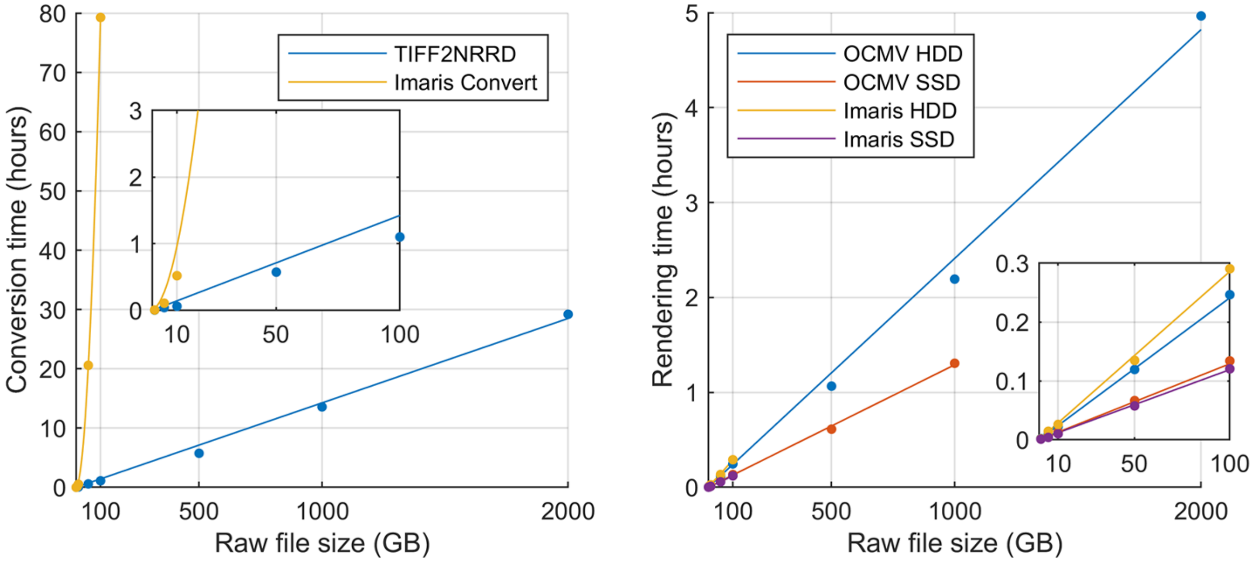

(Left) A directory of TIFF images can be converted to NRRD volumes (for use with OCMV) in linear time, easily scaling to 2 TB datasets and beyond. Context: Imaris File Converter (for converting data to Imaris File Format) exhibits quadratic runtime as a function of input data size, making datasets larger than 100 GB infeasible. **(Right)** OCMV opens and plays live sequences of volumes at a similar rate to Imaris, despite accessing raw data instead of HDF-formatted Imaris files. Operating from an HDD, OCMV outperforms Imaris, while operating from an SSD, Imaris performs slightly better. The complete data are shown in **Supplementary Table 2**.

Supplementary Fig. S4: Navigating two timescales in one timeline

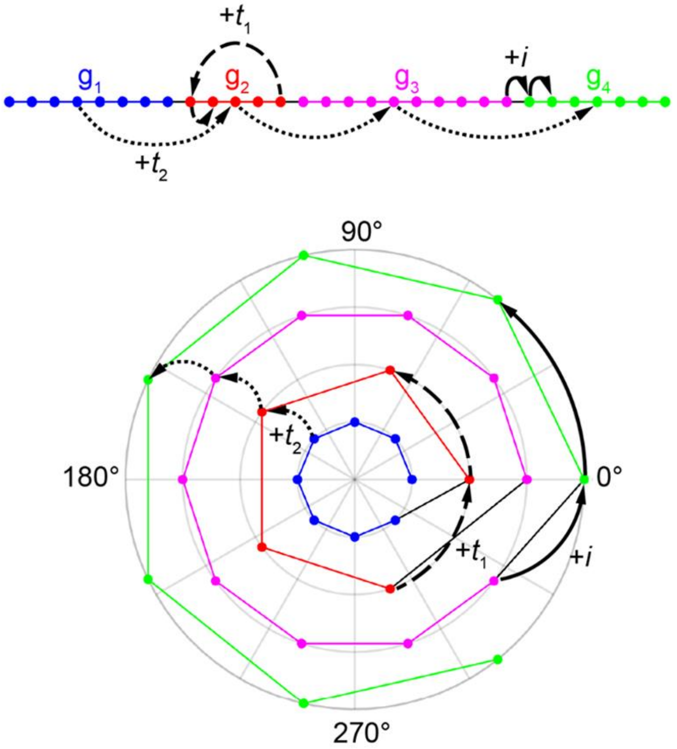

OCMV flattens two timescales onto a single time-axis, sorting by “slow” time-axis (t_2), then by “fast” time-axis (t_1), such that t_2 remains constant within a “group” of timepoints. (Top) Example, g_1, g_2, g_4, and g_4 representing four cycles of a beating embryonic heart at four developmental times (t_2), where each dot is a timepoint (t_1) resolving the heartbeat. (Bottom) These groups can then be considered as rings in a polar coordinate system where angle represents phase of the cycle (t_1), and radius represents developmental time (t_2). Various forward navigation operations in OCMV are shown. The backward operations are simply in the reverse direction. $+t_1$: A step along the fast time-axis, e.g., within a group, wrapping around at the end. $+t_2$: A step along the slow time-axis, e.g., from one developmental time point to the next, maintaining phase as closely as possible. $+i$: A step in the sense of 1D time, ignoring group boundaries. A demo of controlling visualization across two timescales is shown in **Supplementary Video 2**.

Supplementary Fig. S5: Example of an NRRD header file

```
NRRD0005
encoding: raw
type: uint8
dimension: 4
sizes: 2 600 600 252
kinds: 2-vector space space space
labels: "channel" "x-galvo" "depth" "y-galvo"
space dimension: 3
space units: "microns" "microns" "microns"
space directions: (1.635,0,0) (0,1.95,0) (0,0,4.4592)
space origin: (-490.5,-772.2,-558.90498)
data file: G07_T53.raw
acquisition date:=08/30/2016
time index:=24
n times:=80
timestamp:=215 min
period:=0.01
period unit:=sec
group index:=7
```

A detached header for an 8-bit, 2-channel volume image including all the non-standard fields parsed by OCMV (*time index*, *n times*, *timestamp*, *period*, *period unit*, and *group index*), along with some of the recommended fields detailed in **Supplementary Note 3**, and an example of a user-defined *acquisition date* field that will be ignored by OCMV, yet nonetheless improves human-readability of the data file.

Supplementary Fig. S6: Text-based (JSON) scene files corresponding to the GUI

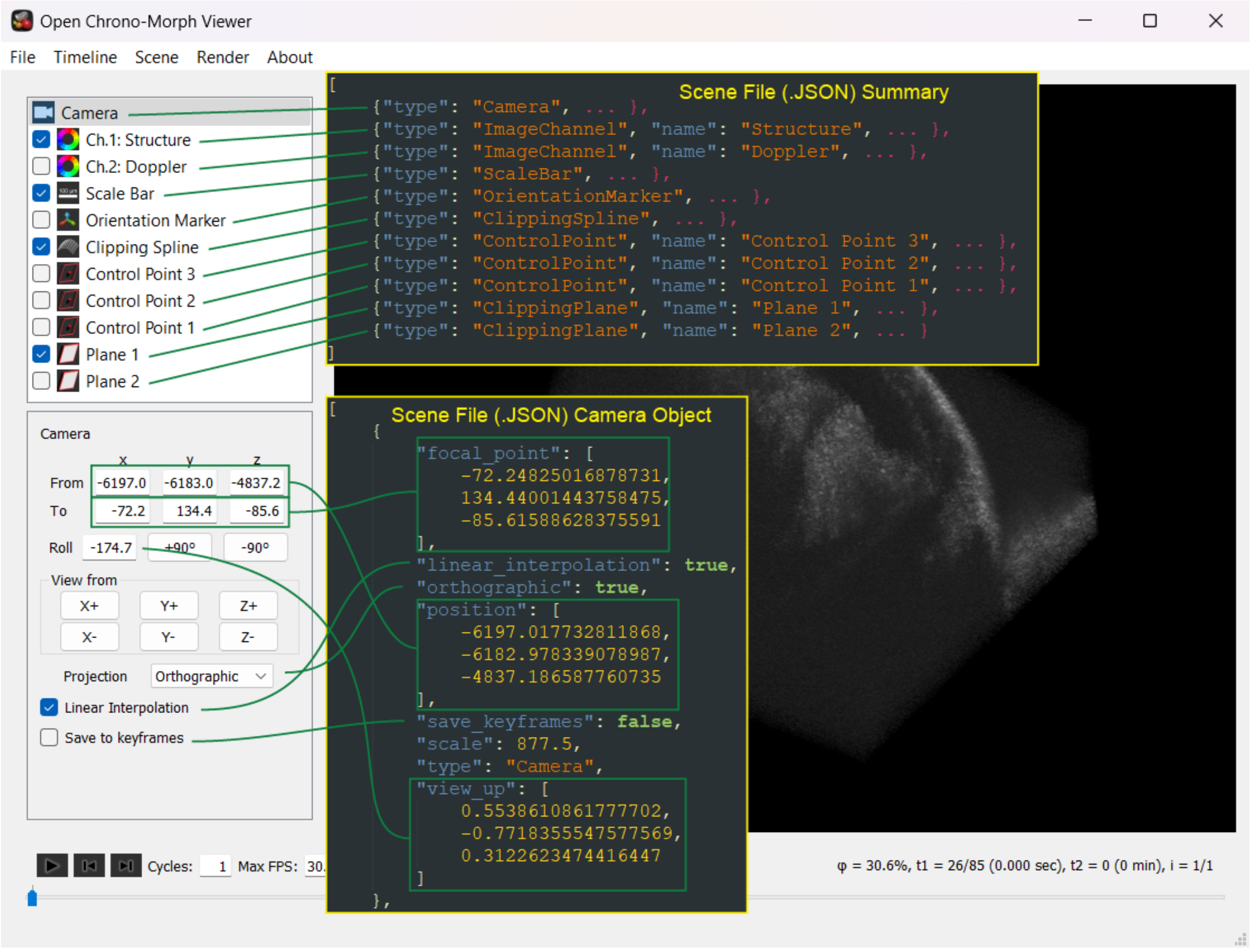

All the scene items in OCMV are quantitatively specifiable and can be exported to scene files in JSON format. Items in the scene list (top left) correspond to objects in the scene file. These items are shown abbreviated by their type and name (upper right). Each scene object in the file contains fields (lower right) that closely correspond to those shown in the scene item panel (lower left).

Supplementary Table 1: Heterogeneous volumes in one timeline

Group	Timepoints	Voxels per Volume	Group	Timepoints	Voxels per Volume
1	85	84,240,000	13	68	104,760,000
2	80	89,640,000	14	68	106,200,000
3	80	90,360,000	15	68	106,920,000
4	80	90,000,000	16	66	108,360,000
5	80	90,360,000	17	65	109,800,000
6	80	89,280,000	18	66	108,360,000
7	80	89,640,000	19	65	110,520,000
8	80	90,720,000	20	65	110,880,000
9	75	94,680,000	21	65	111,960,000
10	75	95,760,000	22	63	112,680,000
11	73	98,640,000	23	63	115,200,000
12	71	101,160,000	24	61	118,440,000

This example shows the voxel number of each group in the OCMV timeline for **Supplementary Video 2**. The groups (slow timescale) contain different numbers of timepoints (fast timescale) and different voxel numbers in each volume because of how 4D alignment was performed. This dataset can be directly visualized by OCMV without resampling, as shown in **Supplementary Video 2**.

Supplementary Table 2: Performance on large sequences of volumes, full data

OCMV / TIFF Convert		HDD				SSD		
**Volumes (#)**	**Data size (GB)**	**Conversion (s)**	**Open file (s)**	**Render all (s)**	**Sum (s)**	**Open file (s)**	**Render all (s)**	**Sum (s)**
3	1.06	14.929	2.518	5.005	7.5	1.524	3.036	4.6
14	4.93	152.869	2.491	31.800	34.3	1.812	20.113	21.9
53	10.06	224.712	1.876	79.105	81.0	1.433	46.219	47.7
274	50.14	2068.017	3.029	426.729	429.8	4.058	235.414	239.5
562	100.01	3967.092	4.243	882.778	887.0	7.64	474.51	482.2
1248	500.40	20679.166	9.185	3826.04	3835.2	16.57	2186.95	2203.5
2866	999.94	48858.579	48.940	7846.581	7895.5	33.67	4669.36	4703.0
6796	1999.92	105085.755	81.781	17794.700	17876.5			
Imaris / Imaris File Converter		**HDD**				**SSD**		
**Volumes (#)**	**Data size (GB)**	**Conversion (s)**	**Open file (s)**	**Render all (s)**	**Sum (s)**	**Open file (s)**	**Render all (s)**	**Sum (s)**
3	1.06	23.962	1.87	4.19	6.1	1.43	2.40	3.8
14	4.93	388.051	3.88	48.07	52.0	1.87	12.65	14.5
53	10.06	1873.413	5.63	88.17	93.8	2.03	34.24	36.3
274	50.14	74076.203	15.63	469.77	485.4	2.86	204.36	207.2
562	100.01	285356.227	28.19	1017.29	1045.5	4.09	429.61	433.7
Imaris raw measurements		**HDD**						
**Volumes (#)**	**Data size (GB)**	**Open file (s)**			**Render all (s)**			
3	1.06	1.78	1.53	2.29	4.18	4.20	4.18	
14	4.93	3.03	4.93	3.69	50.11	47.48	46.63	
53	10.06	4.80	5.15	6.95	88.13	88.51	87.88	
274	50.14	15.98	15.38	15.53	468.41	465.65	475.26	
562	100.01	28.22	28.01	28.35	1019.36	1025.29	1007.23	
		**SSD**						
**Volumes (#)**	**Data size (GB)**	**Open file (s)**			**Render all (s)**			
3	1.06	1.28	1.37	1.65	3.08	1.87	2.25	
14	4.93	1.49	1.78	2.33	12.87	12.87	12.21	
53	10.06	2.56	1.78	1.74	34.04	34.36	34.33	
274	50.14	3.06	2.90	2.61	204.43	207.66	200.98	
562	100.01	4.11	4.34	3.81	429.75	434.14	424.94	

Experimental processing time for file conversion and playback in seconds as a function of the raw voxel data size in gigabytes. “Conversion” represents the time for the appropriate converter to produce the requisite file format from a directory of TIFF images, i.e., NRRD via *tiff2nrrd*, a script for OCMV, and “.ims” via Imaris File Converter for Imaris. The “Volumes” column indicates the corresponding number of volumes (each containing 600×600×~450 voxels and 2× 8-bit

channels) converted from the TIFF stack. “Open file” represents the time from selecting a file to open to the first volume being rendered, excluding automatic channel adjustment in the case of OCMV, and subsequent image refinements in the case of Imaris. “Render all” represents the time from the first volume to be rendered until the last volume has been rendered. “Sum” represents both the opening and rendering times together. The original stopwatch measurements for the Imaris file opening and rendering times are provided under the header, “Imaris raw measurements”. The summary plot is shown in **Supplementary Fig. S3**.

Supplementary Table 3: Comparison with existing volumetric time series software

	**OCMV** (this paper)	**Imaris**	**napari**	**Icy**	**ImageJ**	**Vaa3D**
1. Version tested	1.0	9.6.0	0.5.4	2.5.2	1.54f	x.1.1.4
2. Language	Python	C/C++	Python	Java	Java	C/C++
3. Availability	5, Open source	1, Commercial	5, Open source	5, Open source	5, Open source	5, Open source
4. 3D visualization tools and rendering capabilities	4, Many common 3D clipping functions and accessories, some rigidity in workflow	3, Good clipping tools, limited control of the transfer function	2, Some 3D rendering capabilities, limited clipping tools without scripting	3, One clipping plane, good transfer function specification	1, Limited 3D rendering and video playback capabilities with few functions	2, Some 3D rendering capabilities focused on ortho slicers
5. Animation support and usability	3, Full control via scripting interface, requires knowledge of Python	4, UI keyframe interpolation which is capable, but difficult to use and modify	3, None built in, scripting is possible with Python	2, UI keyframe camera animation but no volume clipper control	1, Time playback exclusively	2, Simple rotation presets only
6. Reproducibility and state saving	4, Saves viewing state to file, lacks undo/redo	3, Saves some viewing state, but not the camera position	2, Saves no viewing state without scripting in Python	3, Saves selections in XML for each image, but no 3D viewer state info	1, Saves almost no viewing state	1, Saves almost no viewing state
7. Multiple timelines	4, Two interleaved timelines, heterogeneous volumes permitted	2, One timeline, homogeneous volumes only	4, Unlimited distinct timelines, heterogeneous possible, but difficult	2, One timeline, homogeneous volumes only	2, One timeline, homogeneous volumes only	2, One timeline, homogeneous volumes only
8. Big data file support	5, Directly opens multiple files	3, Must convert, slow when large, single file only	4, Available through scripting or for specific file formats	4, Directly opens multiple files, cache manager is in beta	2, Limited capabilities	2, Must convert, very slow, rigid directory structure only
9. Stability on Windows 10/11	4, Main functions work reliably, some bugs, logs errors	1, Repeated fatal errors and buggy functionality across a broad swathe of use cases	4, Main functions work reliably, some bugs	4, Reports errors but doesn't crash, shipped with non-functional VTK library	5, Mostly works, catches the rare exceptions, almost never crashes	3, Crashes during certain operations, some bugs
10. Ease of use	4, Simple interface, animation requires scripting	3, Reasonably intuitive, but several broken behaviors with complex workarounds	2, Simple UI but requires knowledge of Python for most useful behaviors	5, Very clean interface, few bizarre behaviors	4, The basics are simple and well documented, many plugins are unintuitive or poorly documented	3, UI is intuitive, but documentation is outdated, and time series handling is difficult
11. Data functions, analysis, ROIs, markers, and annotation	1, None exist	5, Rich data processing and analytical capabilities	4, Access to the full suite of Python tools, but programming is required for most	5, Rich data processing and analytical capabilities, bundles ImageJ	5, Rich data processing and evaluation capabilities	4, Rich data processing focused on neural tracking among others

A compilation of the author's opinions qualitatively comparing the functionality of several popular volumetric visualization platforms based on subjective user experience for inspecting volumetric

time series images on Windows 10 and 11. Capabilities for each metric were rated on a 1-to-5-point-scale, where 5 is the best, and colored as follows: orange for 1–2, yellow for 3, and blue for 4–5. The nine comparison criteria were chosen to highlight OCMV's unique qualities and use cases. Additional analyses are provided in **Supplementary Note 9**.

Descriptions of Supplementary Videos

Supplementary Video 1: Tutorial about scenes and saving keyframes for animation

A tutorial demonstrating how to save a scene file to store a visualization configuration for later, load a saved configuration, edit scenes, and save certain scene elements to keyframes for animation. This tutorial includes audible dialog.

Supplementary Video 2: Tutorial about the timeline interface

A tutorial demonstrating the layout of the volumes in the timeline and the various controls for navigating the two timescales. This tutorial includes audible dialog.

Supplementary Video 3: Demonstration of animation editing

A video showing animation scripts and how they can be modified to produce different animation effects, including channel intensity correction, camera motion, movement of clipping planes, speeding up, reversing time, and adding text overlays. Data from Zenodo (https://doi.org/10.5281/zenodo.6448019) under CC BY 4.0 license.

Supplementary Note 1: Installation instructions

OCMV requires a standard installation of Python, along with some 3rd party libraries. Animation frames can be made without FFmpeg, but they cannot be compiled into a video file through OCMV without FFmpeg installed and added to the environment variables.

This installation process should take 5–10 minutes.

Required software

- Python version 3.11.4
- FFmpeg version 6.1.1 (only required for animating videos)

Required 3rd party Python packages

- NumPy 1.25.2 for 3D data operations
- PSutil 5.9.5 for getting the current RAM usage
- PyNRRD 1.0.0 for the image file format (Nearly Raw Raster Data)
- PyQt5 5.15.2.2.3 for the user interface
- PyVTK 9.2.6 for volumetric rendering
- SciPy 1.11.1 for the "cdist" function used in the clipping spline
- TIFFfile 2023.7.18 for parsing TIFF files

Windows 10/11

A binary distribution is available for Windows. If you would like to use the binary version, just download it from the GitHub:
https://github.com/ShangWangLab/OpenChronoMorphViewer/releases/tag/Public

… then extract it anywhere. If you merely want to view files, you may stop here. However, the binary distribution cannot be used to write animations scripts; for those, you must continue to install the Python requirements along with FFmpeg.

Install Python with dependencies:

1. Download and open the latest version of the Python installer from https://www.python.org/downloads/windows, found under “Download Windows installer (xx-bit)”. This should usually be the 64-bit version. Python version 3.10+ is required, while 3.11 is the best-tested version.

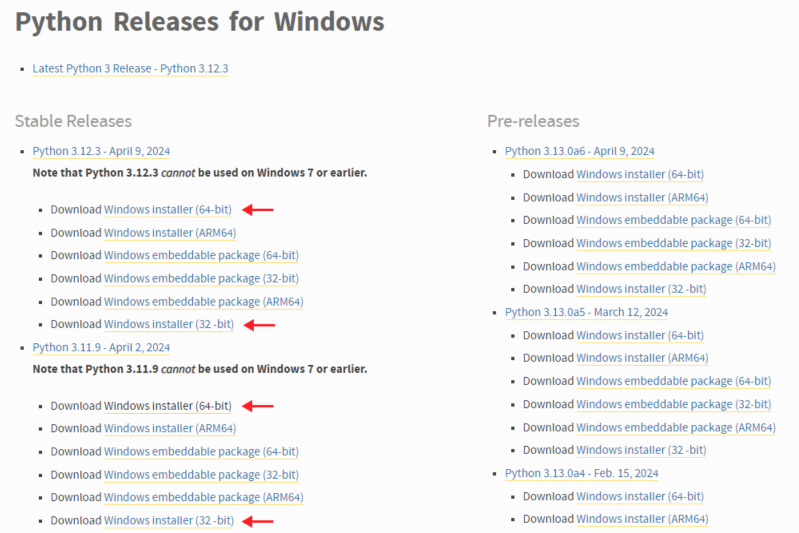

2. Check the box to add python.exe to PATH. If you already have an installation of Python, this may cause issues with overlapping installation names.

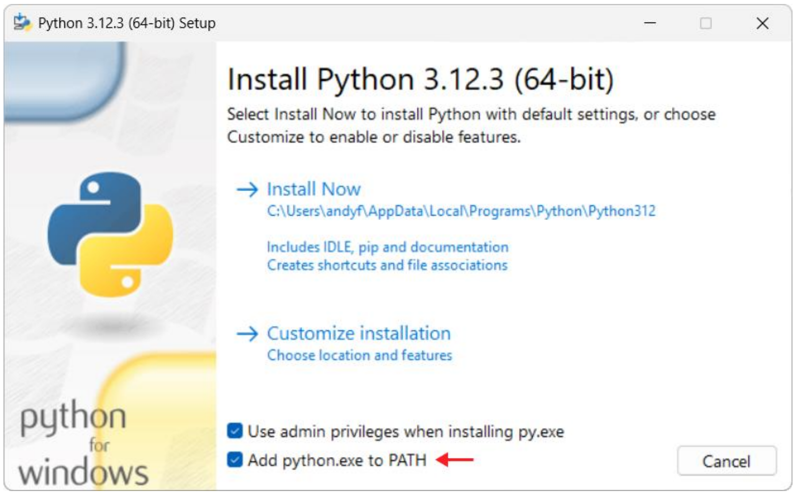

3. "Click Install Now" or make a custom installation. If the latter, make sure not to uncheck the box to also install pip, and do not uncheck the option to add Python to the environment variables.
4. Clone the OCMV GitHub repository (https://github.com/ShangWangLab/OpenChronoMorphViewer) to your computer and extract the ZIP file, if necessary.

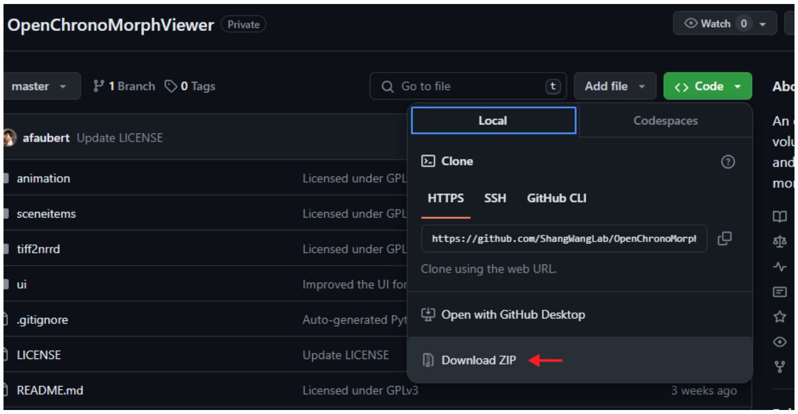

5. Run “dependency-install-Windows.bat” to install the required Python packages using pip.

Install FFmpeg (optional):

1. Go to https://github.com/BtbN/FFmpeg-Builds/releases and download “ffmpeg-master-latest-win64-gpl.zip”.

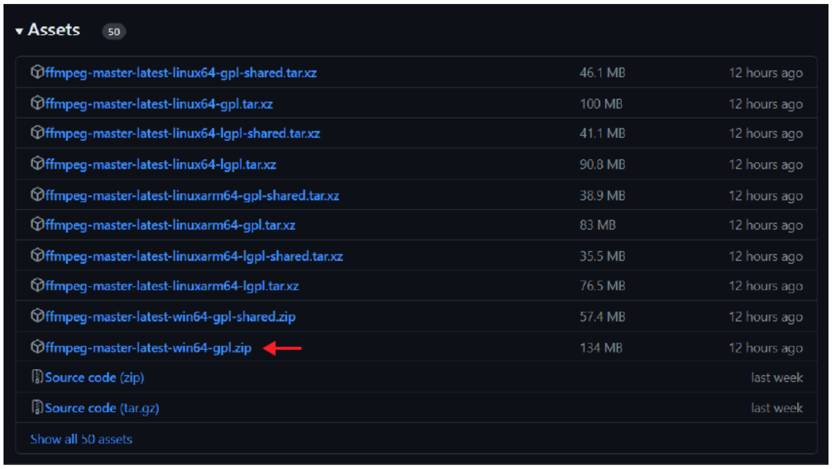

2. Extract the zip somewhere permanent, like, “C:\Program Files\ffmpeg\”.
3. Add the bin directory to your path: “Edit the system environment variables” in the Start menu (under “Settings/About/Advanced system settings”), select “Environment Variables…”, double-click on “Path” for either the User or System variables, and add an entry for the “bin” subdirectory, e.g., “C:\Program Files\ffmpeg\bin”.

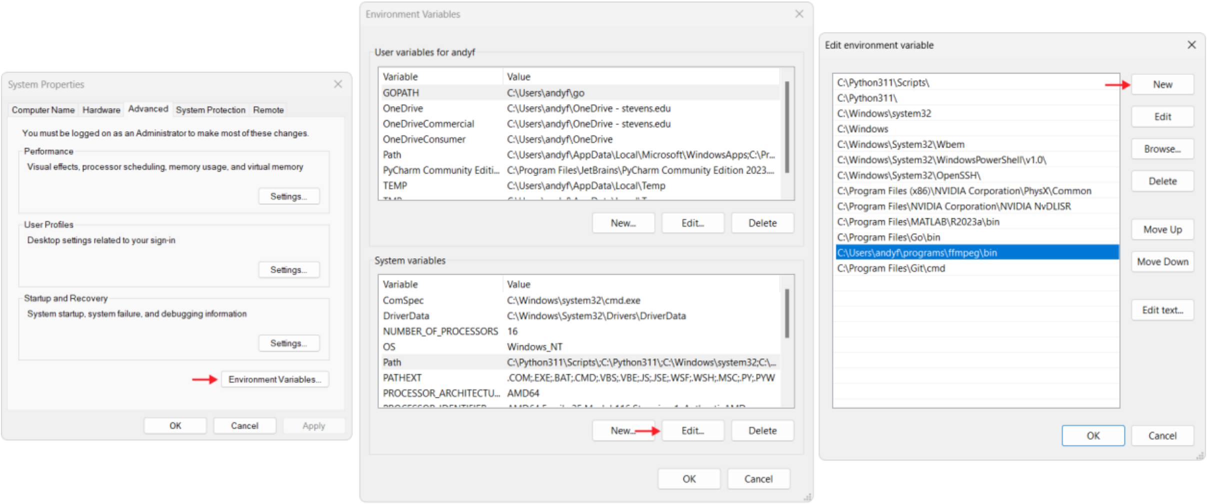

Run OCMV:

1. Open the OCMV repository previously downloaded and double-click on “run-OCMV-Windows.bat” to start the software.

macOS 13.0+

Install Python with dependencies:

1. Install the latest version of Python from https://www.python.org/downloads/macos, found under “Download macOS 64-bit universal2 installer”. Python version 3.10+ is required.

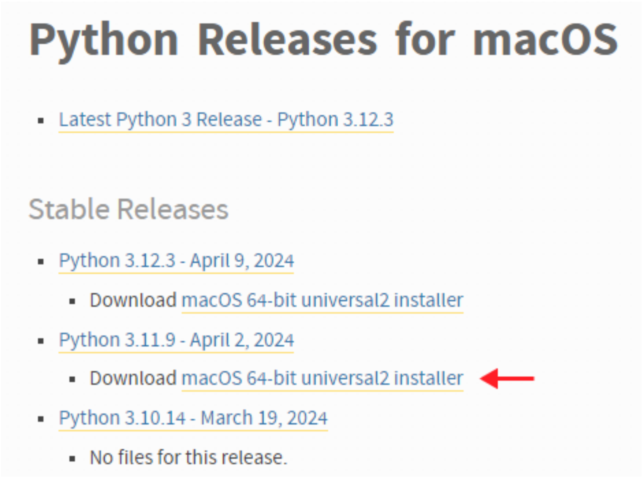

2. Double-click the installer to install Python. If you already have an installation of Python, this may cause issues with overlapping installation names.
3. Run “Update Shell Profile.command” under the new installation directory by double-clicking it.

4. Clone the OCMV GitHub repository (https://github.com/ShangWangLab/OpenChronoMorphViewer) to your computer and extract the ZIP file, if necessary.

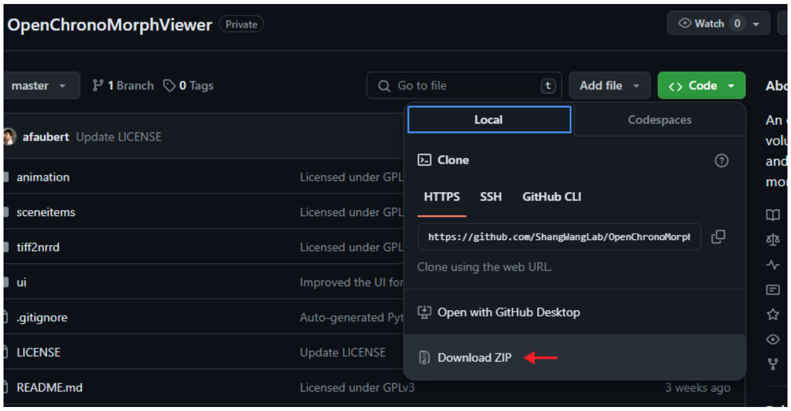

5. Open a terminal and navigate to your OCMV folder using the “cd” command.

```
cd ~/Downloads/OpenChronoMorphViewer-master/
```

6. In the terminal, use “chmod” to acquire permission to execute the OCMV files.

```
chmod +x *
```

7. In the terminal, run “./dependency-install-macOS.command” to install the required Python packages using pip.

Install FFmpeg (optional):

1. Go to https://evermeet.cx/ffmpeg and, under “ffmpeg-x.x.x.7z” on the right, click “Download as ZIP”.

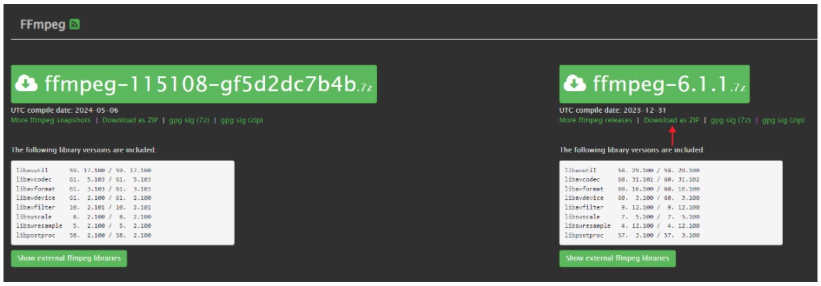

2. The ZIP file should contain a single executable called “ffmpeg”. Extract the ZIP file and move ffmpeg to “/usr/local/bin/”.

Run OCMV:

1. Open the OCMV repository previously downloaded and double-click on “run-OCMV-macOS.command” to start the software.

Ubuntu 20.04+

Install Python with dependencies:

1. Open a terminal.
2. Add the deadsnakes PPA, which provides specific version builds of Python for Ubuntu.

```
sudo add-apt-repository ppa:deadsnakes/ppa
```

3. Now that Python version 3.11 can be located, install it.

```
sudo apt-get install python3.11
```

4. Install the correct version of the Python package manager, PIP.

```
wget https://bootstrap.pypa.io/get-pip.py
sudo python3.11 get-pip.py
rm get-pip.py
```

5. Install the required Python libraries. Sometimes, an existing library will cause installation issues, so we recommend forcibly reinstalling them.

```
python3.11 -m pip install --force-reinstall numpy psutil pynrrd pyqt5
scipy vtk
```

6. Clone the OCMV GitHub repository (https://github.com/ShangWangLab/OpenChronoMorphViewer) to your computer and extract the ZIP file, if necessary.

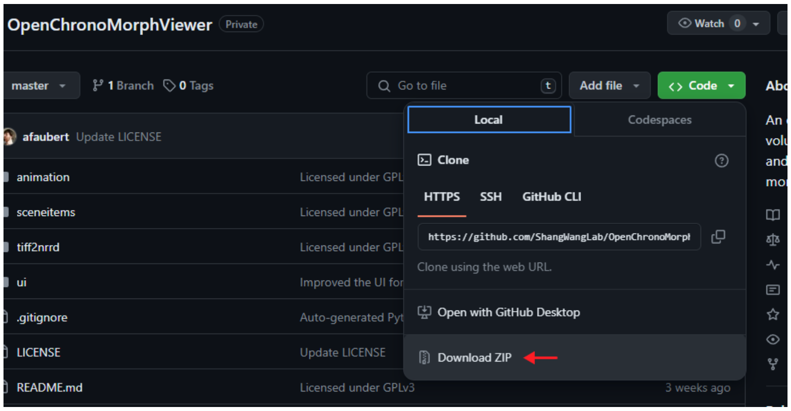

Install FFmpeg (optional):

1. FFmpeg usually comes prepackaged with Ubuntu repositories. If it is missing, you can install it with apt-get.

```
sudo apt-get install ffmpeg
```

Run OCMV:

1. Navigate a terminal to the OCMV repository previously downloaded and run:

```
python3.11 openchronomorphviewer.py
```

Supplementary Note 2: Overview of the graphical user interface

OCMV's graphical user interface (GUI) is for real-time, interactive control of visualization and saving of keyframes/scenes.

Viewport controls

The viewport controls are inherited from VTK's trackball camera interactor. The controls are as follows:

Orbiting about the focal point can be performed by clicking and dragging with the left mouse button.

Panning can be performed by holding the middle mouse button and dragging or by holding Shift and dragging with the left mouse button.

Spinning around the axis of the viewport can be performed by holding Ctrl and the left mouse button and dragging in a circle.

Zooming in or out can be performed by holding the right mouse button and dragging up and down for in and out, respectively, or by holding Ctrl and Shift simultaneously while dragging up and down with the left mouse button.

Menu bar functions

The menu bar contains a variety of functions. Each function is self-documented in OCMV via a tooltip which shows upon hovering the mouse over the menu button. Many of these functions have an associated keyboard shortcut, and in fact, all valid keyboard shortcuts except for the viewport controls have an associated menu item. If a shortcut exists, it will be displayed to the right of the function's title in the menu bar.

1. File
    a. Open Volumes… Open a menu to select and load a selection of NRRD files.
    b. Save Scene… Write all settings and visual elements to the last "scene" file. When no file has previously been saved, this is equivalent to Save Scene As…
    c. Save Scene As… Open a menu to select a file for saving the "scene" to.
    d. Save Keyframe Save the selected subset of the visual elements in the scene to a "keyframe" file. The subset is determined by the checkboxes labeled, "Save to keyframes".
    e. Load Scene… Open a menu to retrieve the "scene" from a file of your choice, updating the visual elements and settings.
2. Timeline
    a. Play/Pause Autoplay or stop autoplaying volumes.

b. Next Volume — Move the timeline slider right by one step.
c. Prev. Volume — Move the timeline slider left by one step.
d. Next Group — Jump to the next volume group.
e. Prev. Group — Jump to the previous volume group.
f. Start of Group — Jump to the first volume of the current group.
g. End of Group — Jump to the last volume of the current group.

3. Scene
   a. Delete Item — Delete the active scene item, if possible.
   b. Deselect Item — Hide the current scene item controller by deselecting it.
   c. Toggle Item — Check/uncheck the active scene item.
   d. Look at Plane — Move the camera so it is normal to the active plane.
   e. Plane to View — Turn the active plane so it is normal to the camera.
   f. Adjust Channels — Set the channel ranges using the histogram from the current volume.
   g. Place Control Point — Add a new control point underneath the cursor position. It is recommended to use the keyboard shortcut rather than this menu item.
4. Render
   a. To Image… — Save the current viewport as an image file. The options are PNG, TIFF, and JPEG.
   b. Settings… — Edit settings related to the render frame, in particular, this allows a fixed viewport size to be set for rendering images of a certain dimension.
5. About
   a. Project… — Open a web browser to the OCMV project page on GitHub.
   b. Acknowledgements… — Display the acknowledgements.
   c. Version… — Display the current version number of the software.

Scene items and timeline

This section describes the user interface as shown in **Fig. 1**.

Objects which control how the volumes are displayed are called “scene items” and are stored in the scene list (a). When a scene item’s check box (b) is checked, that scene item will affect the visualization. You can click on a scene item to select it, which will cause it to become active and enter its information into the scene panel (j). Here, a clipping plane (c) is selected. Certain items, like clipping planes, will cause visible controls (e) to appear inside the volume (d) when selected. These controls can be used to quickly manipulate the scene item. Scene items can also be quantitatively modified in the scene panel, which contains all the user-editable parameters of the item.

A clipping plane includes a "title" box where the display name can be edited for book-keeping purposes, buttons to create a new plane or delete an old one (f), controls for setting the XYZ values of the plane's origin and normal vector (g), and buttons to "look at" the plane, i.e., change the camera to view normal to the plane, or make the plane "normal to view" to change the plane to match the camera (h). All scene items contain a "save to keyframes" (i) checkbox which, when checked, causes that scene item to be exported when you save your next animation keyframe.

While only the selected scene item is displayed in the lower left panel, views for the other items have been arranged along the bottom for illustrative purposes. The camera item (s) controls how the current volume is projected onto the viewing plane. It includes the origin of the camera ("from"), the focal point of the camera ("to"), the roll of the camera relative to the positive y-axis, buttons to snap the view normal to a particular axis, a projection mode, which can be either orthographic or perspective mode, and a checkbox controlling linear vs. nearest sample interpolation.

A volume can have up to four intensity channels. Each channel is associated with a scene item controlling how the numeric data of the volume maps onto the color on the screen. The channels can be named for convenience. You can add a new channel, up to four, or you can delete an existing channel. There are two transfer functions available: linear and triangular. The opacity range and dynamic range are interpreted differently depending on the transfer function. Opacity represents the height of the line in the transfer function visualization (t) as a function of image value, and controls how opaque a voxel is to rays projecting through it, while the dynamic range sets the left and right thresholds between which, colors are interpolated, and outside of which, colors saturate. The linear transfer function (shown) maps values between the minimum and maximum of the image value linearly to a "minimum" and "maximum" color, and to the opacity value of each voxel. The center color is ignored when in linear mode. Triangular mode is used for representing positive and negative values. The central image value becomes the low opacity value, with opacity increasing linearly to the left and right, and colors approaching the "low" and "high" colors. The color codes are shown on the right of the color selector buttons, which also show a tile of the selected color.

The scale bar causes a line of known width to be drawn in the lower left (n) while in orthographic projection mode. The width can be set in both microns ("width") and in screen pixels ("zoom"). Setting the number of screen pixels causes the camera zoom to change such that the width in microns and pixels match. The size label can be shown or hidden with the "show label" checkbox. Units are automatically calculated from the micron width scale entered.

The control points define the clipping spline. Three points are automatically created the first time the clipping spline is checked. You can edit the label for convenience, add a new

control point in a random location, or delete a control point. The origin of the control point is used as its location in space, while the normal specifies the plane normal of the control surface used for moving the control point but does not affect the resulting spline. When the scene item's box is checked, a green sphere is shown at the origin (o). Like a clipping plane, the camera can "look at" the plane, or the plane can be made "normal to view". When selected, a plane controller will appear in the viewport, allowing you to quickly manipulate the control point.

The clipping spline (p) causes a smooth surface to be cut away from the volume, passing through each control point. "Variable" controls which direction the spline cuts from, along with which side to cut away. When the left-hand side of the equation is less than the right, the lower end of the axis is cut away, and vice versa. When smoothing is positive, some deviation from the control points is permitted to minimize the curvature of the spline. In the limit as "smoothing" approaches infinity, the clipping spline becomes a planar fit to the control points. "Up-scale" speeds up mask generation by creating the mask with proportionally fewer voxels and projecting the upscaled version onto the volume. Thus, the speed-up is proportional to this factor cubed: one multiple for each of the three spatial axes. However, the larger the upscale, the coarser the resulting block pattern on the surface. The "show mesh" checkbox causes a yellow grid to be shown on the volume, indicating the surface of the clipping spline.

The orientation marker (q) shows the orientation of the view relative to the principal axes of the volume's coordinate system. The marker can be adjusted by clicking and dragging its bounds, or the size can be adjusted with the "width" box.

The timeline slider (k) controls which volume to display. Volumes are selected via the "File" menu, and automatically laid out in the timeline by group. Group boundaries are shown as alternating light and dark grey regions. The timeline controls (l), from left to right:

1. The "play" button causes the timeline to automatically advance from one volume to the next.
2. The "previous volume" button takes you back one volume, while taking cycle looping into account.
3. The "next volume" button steps to the next volume, likewise, accounting for looping.
4. The cycle counter box causes the playback to loop that number of times before advancing from one volume group to the next, or indefinitely when set to zero.
5. The maximum framerate limit box sets the number of frames per second to play back when playback is unbounded by hardware limitations.
6. The "go to" button takes you to the volume index specified.

When idle, volumes are automatically loaded from the disk to the cache until the cache limit (m) has been reached.

The label in the lower right (r) shows volume indices and timing information associated with the currently visible volume, as stored in its NRRD file. From left to right:

1. "φ" displays the cycle phase as a percentage.
2. "t1" shows the absolute time index within a cycle, or volume group, out of how many indices are in the cycle, and the physical time in parentheses.
3. "t2" shows the absolute group index and the physical time in parentheses.

"i" shows the absolute volume index out of the entire timeline.

Supplementary Note 3: The NRRD file format and its benefits

Comparison between TIFF and NRRD

For decades, Tagged Image File Format (TIFF) has been the standard in the bioimaging community, championed by tools such as Fiji, a selection of plugins packaged with the popular image analysis software, ImageJ. A TIFF file represents a binary image, or list of images, each with a set of numeric tag IDs and corresponding binary values for each tag. Early versions of ImageJ supported only simple stacks of images, but in 2007, version 1.39g introduced hyperstacks, allowing up to 5D datasets to be represented in a single file, including the time- and channel-axes along with the existing stack Z-axis. While the Nearly Raw Raster Data (NRRD) file format (https://teem.sourceforge.net/nrrd/index.html) has existed for decades and exists as a distribution format for certain public projects, it has remained obscure, at least in part due to a lack of available tools to manipulate its files. An NRRD file consists of a plain-text header containing a list of text tags and text values, along with a block of binary data corresponding to the “raw” raster data of the image array. While TIFF can be thought of as a 3D stack of distinct images, the payload of the NRRD file supports an arbitrary number of axes and should be thought of as an N-dimensional (N-D) scalar array. Thus, the two salient differences between the formats are the binary tags vs. the plain-text tags in the header, and the conceptualization as one or more images vs. an arbitrary N-D array.

Today, the scientific community primarily uses TIFF files to structure multidimensional image data in one of two ways, either as a TIFF stack, storing each image internally, or by relying on the file system, i.e., writing individual TIFF image files to a directory with array indices embedded in the file names. The former method is the technically superior choice, as it allows metadata to be embedded in the file instead of cluttering the file name while also drastically improving the read/write speed over the latter, with its thousands of separate files. However, the fact that the latter method exists at all speaks to a desire for simplicity; specific libraries are required to extract the images from a TIFF stack and parse the binary metadata, adding a hurdle to the challenges of scripting and automated image visualization and processing.

We propose NRRD as an alternative for multidimensional image visualization and processing due to its rare combination of simplicity and flexibility, as highlighted below.

Metadata

Metadata is crucial, especially when sharing data between team members who may not be intimately familiar with the image format. These requirements lead naturally to a conclusion: raw voxel data with a simple, plaintext header for the metadata, in other words, a “nearly raw raster data” (NRRD) file. This defeats the drawbacks of raw data, namely, the lack of information

about the bounds, data type, etc. See **Supplementary Fig. S5** for an example of the plaintext header.

Because use cases vary widely, extensibility is a key property of a universal data representation. For example, since TIFF does not natively support more than three dimensions, any four- or higher dimensional data must be encoded into the metadata in an ad hoc manner. Since TIFF metadata is in a binary format using tag ID numbers to represent the various fields, it may be unclear what a novel field is intended to convey. In contrast, NRRD uses text-based identifiers, allowing at least some degree of interpretability when novel tags are used. As such, NRRD lends itself to extensible, custom software, recording the settings used directly into the metadata for later reference. These continual references and updates to a document's metadata represent the vitality of a living document; data is meant to be used, and NRRD's plain-text header makes that as simple as possible, allowing reading or writing with any text-editing software, even Microsoft Notepad. Furthermore, the NRRD metadata can be detached into a separate header file, also known as operating in "headless" mode. This allows the data and the header to be easily parsed by different programs, and in fact, a raw file can be mapped directly to memory in most languages without the aid of any libraries at all. This means that the data will always be accessible, even in ecosystems lacking supporting libraries.

In terms of tools for parsing the metadata, both formats are well-supported in two commonplace scientific computing environments: TIFF has a full suite of tools in MATLAB via the "Tiff" package, and in Python via the "tifffile" package on PyPI. Likewise, NRRD has MATLAB support through the Medical Imaging Toolbox ("nrrdinfo", "readnrrd") and Python has support through PyPI ("pynrrd" and "NumPy").

Raster data

While TIFF stacks encode raster data as a list of one or more images, NRRD encodes it exactly as it would be natively laid out in RAM as a multidimensional byte array. This allows the entire array to be "memory mapped", i.e., treated in the code as if it has already been loaded into RAM while the operating system manages which data to keep in RAM, and which to unload. Memory mapping can often improve the performance of processing algorithms while minimizing RAM usage. Since the memory is laid out cohesively, without any breaks in between, it is more straightforward to access slices of the dataset along manifolds other than the original imaging plane without loading the entire image into RAM. This off-axis reading allows large N-D datasets to truly be considered independently from the order in which they were acquired.

While a directory of individual TIFF images is conceptually straightforward and allows simple inspection of the slices via the file browser, this solution is suboptimal for several reasons:

- For data processing algorithms, accessing separate files tends to involve many operating system calls and file seek operations; with a TIFF stack or an NRRD file, the sequential reading plays into the strengths of both spinning rust disks and flash memory alike, typically providing a speed up of an order of magnitude.
- To read data along a slice other than the original imaging plane, every file must be accessed.
- The file system can be slow to iterate over and count the many thousands of file entries.
- Individual images can be easily deleted or moved accidentally, or only partially moved, making the dataset incomplete.
- Index information and other metadata tends to end up being stored in the filename, rendering it difficult to rename all the different files, and providing a disincentive to store all the relevant information due to the strong preference for a short filename.

TIFF supports many different compression methods for pixel data, from simple lossless run length encoding algorithms, to more complex lossless algorithms such as JPEG. NRRD only supports Gzip and Bzip2. However, due to increasingly cheap disk storage, the file size is of little importance, while accessibility and processing speed are essential. As such, we recommend storing data in the raw format, reserving Gzip compression exclusively for archival storage and transmission of files over the internet.

Standard NRRD fields

Beyond specifying the basics—volume dimensions, data type, endian—NRRD headers can contain numerous useful fields. Below, we present some of these standard tags specified in the NRRD format and how they might be used in a practical setting to enhance data communication.

The "content" field can specify a short description of what sample was imaged, allowing researchers to keep the file name short, while fully describing each specimen.

The “labels” and “kinds” fields specify a name for each axis, and how to treat that axis, respectively. Using optical coherence tomography (OCT) as an example, some researchers will choose the direction coaxial with the path of the laser to be second, or “Y”-axis, while others will put it as the third, or “Z”-axis. By labeling these axes appropriately as, e.g., “X galvanometer”, “depth”, and “Y galvanometer”, respectively, this issue can be disambiguated. The “kind” of these three axes should all be “space”, for spatial information. Other kinds include “time”, for one or more temporal axes, “vector”, for arbitrary lists of numbers, and “complex”, for complex-valued image data. One reason to distinguish between space and vector axes is that spatial operations such as a gaussian blur should not be applied between unrelated data types, such

as RGB color values. Again, using OCT as an example, this modality often involves multichannel information, either complex values for the phase and amplitude information from the Fourier transform, or the amplitude (“structure”) and phase (“Doppler”) separately. This distinction can be made both in the “kinds” field as “complex” vs. “vector”, and with labels, explicitly calling out the axis order: “structure, Doppler” for the 2-valued tuple of channels. This is particularly useful for fluorescence microscopy, where multiple channels are often acquired to isolate different labels.

There are three redundant ways to specify sample spacing in the NRRD format: 1) “spacings”, 2) “space directions”, and 3) “axis mins” and “axis maxs”. To resolve the redundancy in the spacing information, OCMV exclusively supports the use of the “space directions” field, since it is the most broadly applicable of the three methods; the “spacings” field cannot be easily used in tandem with origin offset information, while “axis mins” and “axis maxs” has ambiguity regarding the “centers” field.

The simplest way to specify the sample spacing is the “spacings” field, which specifies a distance between each sample along each axis. This, in combination with the “units” field, allows you to define the image scale, potentially using different units for different axes, as might be the case with MRI slices, where the XY-plane is far more densely sampled than the Z-axis.

The more flexible, preferred method is to conceptually separate the axes of the data array from the axes of the physical/temporal space in which they were acquired. To accomplish this, you specify the “space” or “space dimension” fields to describe which coordinate system you are acquiring within, such as “left-posterior-superior”, in the case of medical data, then use “space directions” and “space origin” to describe the matrix transform and origin offset which translate the coordinates of the image array into the world coordinate space. For a simple example of 3D scale and offset, the space directions field would be a 3x3 matrix with axis scale factors along the diagonal, while the space origin would be a 3-vector of scalar offsets. Transforming the array coordinate vector, x_a, into the world coordinate vector, x_w, would then be accomplished as the operation,

$$x_w = \begin{bmatrix} s_x & 0 & 0 \\ 0 & s_y & 0 \\ 0 & 0 & s_z \end{bmatrix} x_a + \begin{bmatrix} o_x \\ o_y \\ o_z \end{bmatrix}$$

Since space directions specify an arbitrary transform matrix, this field can also be used to specify rotation and shear along with just scale, i.e., any affine transformation. In principle, this could be used to acquire many datasets at different scales, orientations, and offsets, then to perform affine image registration to transform the data into the new coordinate system. The ability to specify that transformation in the header allows the researcher to avoid unnecessarily resampling their data into a different coordinate system from the one in which it was acquired.

Note that the dimensionality of the array need not match the dimensionality of the space it is sampled in, as is the case for multichannel images where multidimensional data exists for each point in space and time. Thus, you may have a 5D data array (channel, X, Y, Z, time) which was acquired in the 4D space, “right-anterior-superior-time”, for example.

In addition to “spacings” and “space directions”, there are the “axis mins” and “axis maxs” fields, which can be used to specify the upper and lower bounds of the array in the world coordinate system, thus providing origin and spacing information together. These fields can be ambiguous, since this use of spacing information requires the user to consider whether their samples were acquired in a node-centered or cell-centered manner. As such, these two fields should be used exclusively in tandem with the “centers” field to specify this convention.

Oftentimes, the spacing between image samples is unrelated to the resolution of acquisition system. This can lead to uncertainty as to whether the image is over- or under-sampled. The “thicknesses” field can be used to specify the resolution, or the physical width of the area sampled.

The unit for the scalar values of the image can be given as the “sample units” field. This field is givens as a free-form string, so it is advisable in the case of multichannel data to specify the different units for the different channels, e.g., for OCT, the structure channel may have arbitrary units of intensity, while the Doppler channel may have units of radians/s or mm/s. Except when given as floating-point numbers, oftentimes, the sample values are quantized onto 8- or 16-bit integers, thus losing the unit and scale information. In addition to the “mins” and “maxs” values, which can specify the dynamic range used for visualization, the sample range information can be reintroduced via the “old mins” and “old maxs” fields, which specify the real values corresponding to those upper and lower visualization bounds. For example, an 8-bit quantized Doppler value may be represented on the range 0–255, displayed on the range, “min” = 31 to “max” = 224, which might correspond to the original values of “old min” = -3.08 to “old max” = +3.08, and bear “sample units” of “mm/s”. Retaining this information with the data file makes it easier for unassociated researchers to understand the data.

Non-standard NRRD fields

In addition to the standard NRRD fields, researchers are free to specify arbitrary additional information as non-standard fields. These fields are designated simply by replacing the field-value separator, “: ”, with the “:=” symbol. This allows researchers to use their own internally consistent system to attach additional metadata while keeping the NRRD format parse-able by programs unfamiliar with these non-standard conventions. For example, OCMV uses non-standard fields to specify additional time index, group index, and unit information,

since each volume represents a single timepoint out of potentially multiple temporal axes. See **Supplementary Fig. S5** for an example of the non-standard fields.

Oftentimes, datasets involving many volumes will include a separate Excel spreadsheet or other note file describing the various samples and their characteristics. We believe that the non-standard fields of the NRRD header file make an appropriate location to store these values such that they are always associated with each part of the dataset.

Specific requirements from OCMV

OCMV expects its input data to comprise a selection of separate NRRD files. Each NRRD file represents a volumetric scalar image with one to four channels. The metadata may be included in the same NRRD file as the volume, or in a separate header file (headless mode). In terms of mapping an N-D array onto 1D memory, the channel must be the fastest varying axis in a 4D image file, with the next three axes mapping to the X-, Y-, and Z-axes in decreasing order of variation rate. Alternatively, a 3D image may omit the channel axis, but not the X-, Y-, or Z-axes. Scale information for each spatial axis ("space directions") is required for each volume. The supported pixel data types are currently limited to 8-bit and 16-bit integers. A minimal NRRD header for a 16-bit volume with three channels might look like this:

```
NRRD0005
encoding: raw
type: uint16
endian: little
dimension: 4
sizes: 3 500 1390 100
space dimension: 3
space directions: (0.645,0,0) (0,0.645,0) (0,0,2.58)
```

Here, the space directions consist of three vectors, one for each space dimension. The magnitude of each vector represents the sample spacing for that axis. Presently, rotations and skews in this matrix are not recognized by OCMV, although that may change in the future. Note that "channels" are not considered to be part of the acquisition "space", and as such, do not have an associated "space direction" vector.

Supplementary Note 4: NRRD file conversion

OCMV primarily uses the NRRD file format, while providing limited support for 3D TIFF stacks. Since datasets are not always natively given in these formats, it is essential that one be able to convert between file formats. While a raw file can be "converted" to an NRRD file merely by writing a plain-text header file that refers to it (i.e., a "headless" NRRD file, extension: ".nhdr"), other formats such as a DICOM present a greater challenge. While 3D TIFF stacks may be loaded directly, they do not support some of the additional fields needed to make full use of OCMV.

Fiji—and ImageJ, which it is built upon—represents a popular image processing utility in the scientific community for bioimaging and beyond. In recognition of the paucity of existing image conversion software featuring full support for the NRRD file specification, we have created a plugin for Fiji, building upon the existing Fiji I/O plugin to extend its capabilities from 3D NRRD files up to 5D data, representing multichannel volumetric time-series capabilities. We have implemented the NRRD specification to the full extent possible, with some caveats, such as Fiji's lack of support for images featuring double-precision floating-point numbers, and the lack of representation for hyperstacks of greater than five dimensions, to name a few examples. We hope to merge this plugin with the official build for Fiji. In the meantime, the modified Fiji I/O plugin is available on GitHub (https://github.com/afaubert/IO) and can be installed into Fiji by replacing the existing "IO_" JAR file in the "plugins" directory with "IO_-4.2.3-NRRD.jar" from the GitHub.

Fiji I/O Plugin: NRRD reader and writer

To load NRRD data into Fiji, navigate the menu to "File → Import → NRRD…". This plugin is only capable of handling NRRD files with the axis ordering C, X, Y, Z, T, that is, the channel axis must be the fastest varying, if present, followed by the regular X and Y image axes, then the Z axis for 3D images, and finally, the time axis for time-series. Only the X and Y axes are strictly required to form an image, but if additional axes are present, they must appear in this order. The axis types, C, Z, T, are automatically inferred from NRRD's "space" and "kinds" fields. The scale and offset information contained in the NRRD file is used to set Calibration for the image or image stack, which allows ImageJ to properly report areas, distances, and offsets through the various "measure" functions in the correct units. Virtual stacks are efficiently read from the disk and rewritten to their NRRD file one block of image channels at a time. Additional NRRD fields are recorded in the underlying NrrdFileInfo object attached to the image window and will automatically populate the fields of the NRRD writer, when possible. While all NRRD data types except "block" are supported for reading, only ImageJ's native 8-bit integer, 16-bit integer, and 32-bit floating-point data types may be used for writing. Helpful error messages are

reported when the NRRD file is in any way invalid, allowing the user to correct any mistakes introduced when typing the header data manually.

To compile data into an NRRD file for use with OCMV, gather your data into a hyperstack, then navigate to “File → Save As → NRRD…”. This will cause a dialog box to pop up (shown in the figure below), into which you can enter any additional information you would like to be saved to the header. Alternatively, you can open the saved file in a text editor after writing to edit the header directly. If you choose to take this route, a “headless”, or detached NRRD file is recommended. This can be produced by checking the “Detached header” box in the main dialog. Axis information can be accessed and edited through the “Settings” buttons below. These buttons will only be available for axes which exist in the hyperstack. The “Slice axis” checkboxes can be used to specify a subset of the axes to split into separate NRRD files, one for each sample along the axis, e.g., a 5D hyperstack containing 3D volumes with two channels and 50 timepoints could be written as 2*50 = 100 separate volumes by checking “Slice axis” under both “Channel Settings” and “Time Settings”.

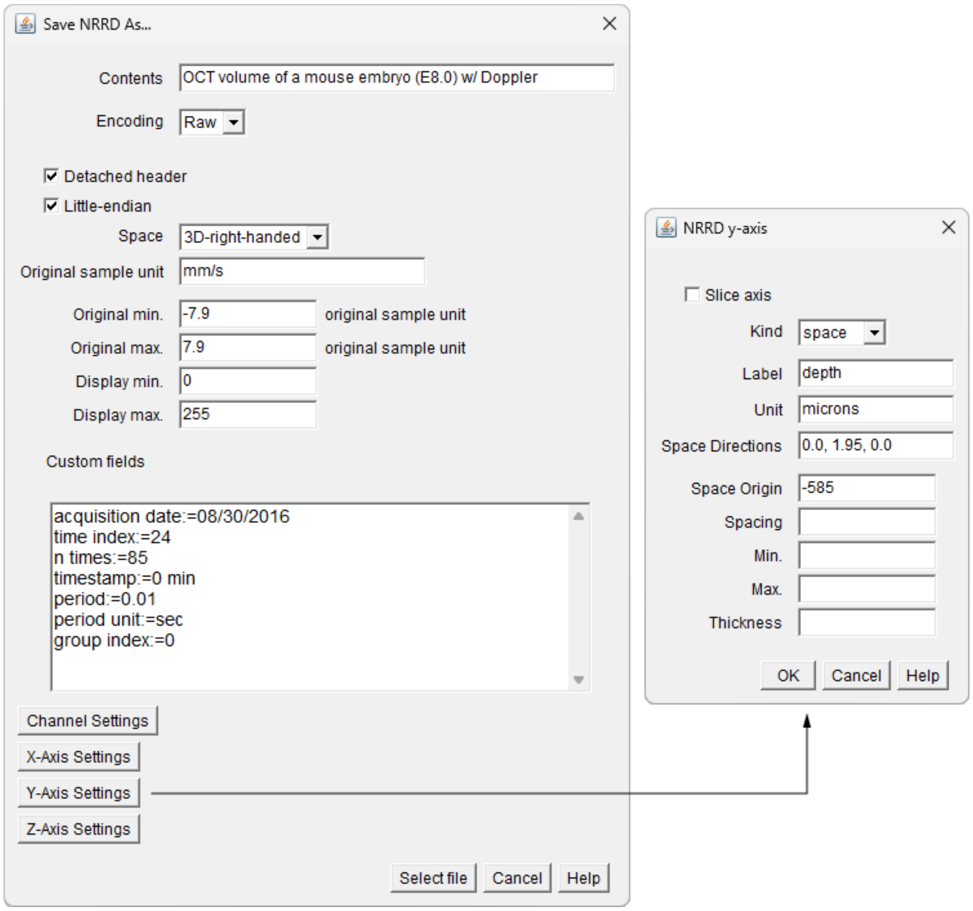

Figure: The NRRD “Save As…” interface from the new Fiji plugin with a multichannel volume hyperstack used as an example. Pressing one of the “Settings” buttons brings up a new dialog with data for that axis.

OCMV requires each NRRD file to contain either a single multichannel volume or a single scalar-valued volume. If your data involves multiple timepoints, OCMV cannot read those timepoints to a single NRRD file. For this reason, the plugin allows you to automatically split a multi-timepoint hyperstack into a series of volume files. To do this, press “Time Settings” (only visible for timeseries) to open the axis settings menu and check the “Slice axis” box. Press “OK” to save the axis settings. When you hit “Select file”, a second dialog will prompt you to choose a name for the file. If you have chosen to split the hyperstack, the split files will automatically bear an index number postfixed onto their file name, along with two non-standard NRRD fields (defined using “:=” instead of “: ” in the header) for each sliced axis, specifying the index of each slice, along with the number of samples that axis contained prior to slicing. These non-standard fields are compatible with OCMV and will be used to automatically arrange volumes into the timeline. When saving volumes individually, it is advisable to use the non-standard fields box to specify these “time index”, and “n times” fields, if applicable. Additionally, the “timestamp”, “period”, “period unit”, and “group index” fields cannot be automatically inferred and will not be written by Fiji. See **Supplementary Note 3** for additional information about these non-standard fields.

tiff2nrrd

Additionally, OCMV comes equipped with a basic, yet quick, file converter found in its installation directory under the title, *tiff2nrrd*. This software can be run from the command line to convert a directory containing individual TIFF, PNG, or JPEG image files into a directory of raw volumes with detached NRRD headers. The command is as follows,

```
python3.11 tiff2nrrd.py <directory of image files> <directory of NHDR files>
```

The images must be named following the form, “name_Ssss_Zzzz_Tttt_Ccc.tif”, where the lower-case letters represent the corresponding digits of the index numbers for the “series” (S), the “time” (T), and the “channel” (C) axes. The order of the axis indices is irrelevant, as are the number of digits used and the starting or ending values for the indices. Errors are not thrown for missing images in between the minimum and maximum indices, instead, these images are left with undefined pixel values. The names of the compiled NRRD files will take the forms, “Sssss_Ttttt.nhdr” and “Sssss_Ttttt.raw”, where the former stores the plain-text header, and the latter stores the raw raster data.

Unlike the new Fiji I/O plugin, the *tiff2nrrd* interface cannot be used to specify voxel scale and origin offset information; that metadata must be added to the NRRD files after their creation. Furthermore, OCMV uses several non-standard fields which cannot be added through this program and must be added to the detached header files afterward if the full capabilities of

OCMV's timepoint grouping are desired. For these reasons, we recommend you use the new Fiji I/O plugin, if possible.

Supplementary Note 5: Adaptive RAM cache management

To view larger datasets than fit into the computer RAM, the data is split by volume into individual files. As illustrated in **Supplementary Fig. S1**, these volume files are dynamically loaded into memory as needed and unloaded when the allocated memory has been filled. To improve performance, nearby volumes—which are likely to be accessed soon—are automatically loaded into cache memory when idle. The prioritization queue is sorted based on proximity to the active (visible) volume:

$$p = \frac{4}{1 + x_f} + \frac{1}{1 + x_b}$$

Where p is the prioritization score of the volume in question, x_f is the distance forward from the active volume to the volume in question, in number of timepoints, and x_b is the distance backwards. Since the volume timeline is a loop, every volume has a distance both in front of and behind the active volume. The forward distance is weighted four times that of the backward distance. For example, if the active volume is at index 71 out of many volumes, the highest priority volumes will be (in decreasing order): 72, 73, 74, 75, **70**, 76, 77, 78, 79, **69**. Here, bold font indicates a volume with lower index than the active one. The additional ones in the denominators act as smoothing parameters and prevent division by zero.

When playing 3D video, playback is I/O-bound, so preloading would harm the playback rate and is therefore disabled. When a new volume must be loaded and the volume cache would otherwise exceed the limit set by the user, the first in, first out principle is followed: the volume(s) which have been accessed the longest ago are unloaded to make room for the new volume.

Supplementary Note 6: Performance on large sequences of volumes

To evaluate OCMV's applicability to large datasets, two aspects were considered: file conversion time from a common format (a directory containing TIFF images), and time to open a file (or set of files) and render the contents to the screen serially. For conversion, we employed a custom Python script called *tiff2nrrd*, which comes packaged with OCMV. We referenced the performance against Imaris 9.6.0 and Imaris File Converter 9.6.0, respectively (Oxford Instruments). Results were acquired on an HP Z4 workstation with a NVIDIA Quadro RTX 4000 graphics card. Processing and conversion times were expected to be I/O-bound, so both an HP 2 TB SATA SSD, and an HP 8 TB 7200RPM SATA HDD were used, with the expectation that the SSD would out-perform the HDD.

The data consisted of large directories of TIFF images to be arranged into 8-bit, 2-channel volumes of approximately 600x600x450 voxels each. OCMV was timed using log files, while Imaris and Imaris File Converter were timed using a stopwatch for an average of three trials (all data provided in **Supplementary Table 2**). "Conversion" represents the time for the appropriate converter to produce the intended file from the TIFF directory (**Supplementary Fig. S3**, left); "Open file" represents the time from selecting a file to open to the first volume being rendered, excluding automatic channel adjustment in the case of OCMV, and subsequent image refinements in the case of Imaris; "Render all" represents the time from the first volume to be rendered until the last volume has been rendered; and "Sum" represents both the opening and rendering times together (**Supplementary Fig. S3**, right). As a practical matter, the "Open file" and "Render all" tests were run on separate instances of Imaris to preclude the preloading of files in the time between opening the file and pressing the play button. Between tests, Windows RAM caches were cleared using RAMMap (Sysinternals) to prevent cache-hits and ensure files were read from the main memory. Due to run-time limitations, Imaris File Converter was only applied up to the 100 GB dataset, while due to limitations on hard drive size, OCMV was only tested up to 1000 GB on the SSD; on the HDD, OCMV was tested up to 2000 GB.

Supplementary Note 7: Overview of the animation API

OCMV includes an application programming interface (API) exposing functions for creating script-based animations.

The goal of animation is to produce a video, i.e., an image that changes over time while the viewing conditions change. In OCMV, an image rendering is specified as some 3D voxel data (the “volume”) in combination with the viewing information that transforms it into a 2D image (the “scene”) (**Supplementary Fig. S1**). You can specify these volumes and scenes for each image in the output video via a Python script.

Basic scripting example

To use the OCMV animation engine, you need to specify where it is installed so that your animation script can find it. This is done by appending the installation path to the environment variables. After adding the path, the *Animator* and Animation Scene (*AScene*) can be accessed.

```
import sys
sys.path.append("C:/.../Open_Chrono_Morph_Viewer/")
from animation.animator import Animator
from animation.ascene import AScene
```

The *Animator* is the main object which manages the animation. It is initialized with the desired framerate of the output video, in frames/s (FPS), and the path to the output directory where individual video frames will be written.

```
anim = Animator(frame_rate=60, dir_out_path="frames/")
```

To load a set of volumes, simply specify the directory containing your NRRD files. You can optionally filter the volumes based on their header information with the *inclusion_criteria* parameter, but here, we want to use all the volumes in the directory. After specifying the volumes, they can be transformed into a series of animation frames by calling *make_frames* on the *Animator* while specifying how quickly the volumes will be played. Here, we specify that 10 volumes will be shown per second. Since our output framerate was set to 60 FPS, each volume will be associated with six consecutive animation frames. The volume rate can also be specified relative to the timing information specified in the NRRD files.

The returned object is called an *AFrameSpan*, which is essentially a list of *AFrame*s bundled into an object with some helpful methods. An *AFrame* specifies a volume to render, a scene to apply to the volume, and, optionally, a list of 2D annotations to add on top, like text or and image overlay. Each *AFrame* will become exactly one frame of the final video.

```
anim.set_volume_dir("volumes/")
frames = anim.make_frames(volume_rate=10, absolute_rate=True)
```

The *AScene*s are loaded from scene files saved from OCMV. These can be either keyframes or regular scenes. Here, we get all our time-invariant viewing parameters from a “main scene” which was saved earlier (**Supplementary Video 1**). Attributes of the scene can also be dynamically adjusted. For example, we set the “scale” of the Camera object to 720 physical units, which are assumed to be microns. In orthographic projection mode, this is half the physical height of the rendered image, for a total height of 1440 microns.

The scene for each *AFrame* in an *AFrameSpan* can be set using the *apply_scene* method.

```
main_scene = AScene.load("keyframes/main_scene.json")
main_scene.items["Camera"]["scale"] = 720
frames.apply_scene(main_scene)
```

To vary a viewing parameter over time, you can either set apply scenes individually using a “for”-loop, or you can interpolate using the built-in methods. The method, *interpolate_camera_1d* allows you to smoothly vary a set of camera positions and orientations over the set of frames it is called upon. The first parameter is the list of relative positions within the *AFrameSpan* to assign to the keyframes specified in the second parameter. Here, there are four keyframes: the camera starts at the default position in the main scene at the start of the span (t=0), moves to the left at t=0.4, shifts to the right at t=0.8, and finishes back at the default position at the end (t=1). These times are specified relative to the length of the span, so t=0.4 would represent 40% of the distance from the start to the end of the frame span.

```
scenes_cam = [
   main_scene,
   AScene.load("keyframes/kf_cam_left.json"),
   AScene.load("keyframes/kf_cam_right.json"),
   main_scene
]
frames.interpolate_camera_1d([0, 0.4, 0.8, 1], scenes_cam)
```

Finally, this set of frames is added to the animator and it is rendered. Rendering the frames causes them to be written to the output directory as images, then those images can be compiled into a video using FFMPEG, called via the command line automatically. The resulting video, example_video.mp4, can be viewed at the end of **Supplementary Video 1**.

```
anim.add_frames(frames)
anim.render_frames(frame_size=(1920, 1080))
anim.compile_video("example_video.mp4")
```

Supplementary Note 8: Reproducing and editing animations

OCMV animation scripts produce videos in a repeatable manner, allowing precise control and iterative refinement of the resulting animation. This note provides several example scripts for the animations shown in **Supplementary Video 3**.

Initial animation script

This basic animation script shows the development of a *Ceratitis capitata* (Mediterranean fruit fly) larva acquired via light sheet microscopy. It shows one pass through the 3D video while interpolating between six different camera positions.

The beginning of the file has the standard imports, specifying the location of OCMV's installation directory, and importing the *Animator* and *AScene* for loading scene files.

```
import sys
sys.path.append("C:/.../Open_Chrono_Morph_Viewer")
from animation.animator import Animator
from animation.ascene import AScene
```

Locations for the volume files are specified, and the location of the save directory for the rendered video frames are specified. The video is set to render in 1080p at 30 frames per second (FPS), advancing the volume counter seven times per second, regardless of any timing information stored in the volumes *(absolute_rate=True*). This produces an *AFrameSpan* object called *frames*, which contains duplicates of volumes to match the 7 volume/sec input specification to the 30 frame/s output specification. One can use *get_optimal_scale* to find the camera zoom level which matches the smallest pixel spacing of the volume set to the size of the viewing window. Since "scale" is half the screen height in physical units, this requires the window height, i.e., index 1 of *frame_size*, to be passed to the function.

```
title = "light_sheet_demo"
volume_files = "C:/.../DS1_4D/"
out_dir_path = f"C:/.../videos/{title}_frames/"
frame_size = (1920, 1080)  # Pixels (wide, high).

anim = Animator(30, out_dir_path, preview=False)
anim.set_volume_dir(volume_files)
frames = anim.make_frames(volume_rate=7, absolute_rate=True)
scale = frames.get_optimal_scale(frame_size[1])
```

The main scene contains all the objects of note, including the scale bar, orientation marker, channel intensity information, and a camera position. The camera keyframes contain a single camera orientation each and can be loaded directly into a list to specify the order of interpolations. The main scene is specified at the end so that the view will conclude while looking from the main perspective.

```
scene_main = AScene.load("scene.json")
scene_main.items["Camera"]["scale"] = scale
kf_cam = [
    AScene.load("kf_cam0.json"),
    AScene.load("kf_cam1.json"),
    AScene.load("kf_cam2.json"),
    AScene.load("kf_cam3.json"),
    AScene.load("kf_cam4.json"),
    scene_main
]
```

After loading the scene and keyframes, they are applied to the video frames. It is important to apply the main scene first to assign it as the scene to use for each frame before interpolating the camera position, which only changes the camera attribute of the scene on each frame. The camera positions are specified in time relative to the length of the video, so 0 means the start of the video, 0.2 means 20% of the way through the video, 1 means the end of the video, etc. The frames are added to the animation, i.e., they will be rendered as part of the video. Frames can also be added to the video more than once to produce repetition in the video.

```
frames.apply_scene(scene_main)
# One set of frames with evenly-spaced camera positions.
frames.interpolate_camera_1d([0, 0.2, 0.4, 0.6, 0.8, 1], kf_cam)
anim.add_frames(frames)
```

Finally, the frames are rendered in whichever order is most efficient in terms of memory management (*keep_order=False*), then compiled into a video using FFmpeg.

```
anim.render_frames(frame_size, keep_order=False)
anim.compile_video(out_dir_path + f"../{title}.mp4")
```

Channel intensity correction

This animation builds on the previous one to introduce the ability to change the channel intensity information to correct a change in intensity across the volume set. As such, we use some notation to provide context: An ellipsis (…) abbreviates some of the code identical to that of the previous script, while a green plus sign at the start of a line indicates the addition of new code.

Multiple interpolations may be applied to the same set of frames. In addition to loading camera keyframes, channel keyframes which specify the intensity values are also loaded. These intensity values are then interpolated between the start, middle, and end of the video using a spline for smoothness.

```
...
kf_cam = [
    AScene.load("kf_cam0.json"),
    AScene.load("kf_cam1.json"),
```

```
    AScene.load("kf_cam2.json"),
    AScene.load("kf_cam3.json"),
    AScene.load("kf_cam4.json"),
    scene_main
]
+ kf_chan = [
+     AScene.load("kf_intensity0.json"),
+     AScene.load("kf_intensity60.json"),
+     AScene.load("kf_intensity120.json")
+ ]

frames.apply_scene(scene_main)
# One set of frames with evenly-spaced camera positions.
frames.interpolate_camera_1d([0, 0.2, 0.4, 0.6, 0.8, 1], kf_cam)
+ frames.interpolate_channels_1d([0, 0.5, 1], kf_chan)
anim.add_frames(frames)
...
```

Text overlay

Text and images can be added in front of an animation frame by adding “annotations” to the frame. To do this, you need to import the *TextAnnotation* class. The font style, size, and alignment of the text’s origin to its bounding box are stored in a *TextStyle* object. The group index of a volume is defined in its file header. A function called *annotation_from_label* defines how to extract the group index of a frame’s associated volume and convert it to the number of developmental hours for a time label in the upper right corner. The right side of the text aligns 99% of the way from the left to the right, and the top of the text aligns 98% of the way from the bottom to the top. This function is applied to annotate all frames and will be reused in later examples.

```
...
from animation.ascene import AScene
+ from animation.annotation import (
+     TextAnnotation,
+     TextStyle
+ )
...
frames = anim.make_frames(volume_rate=7, absolute_rate=True)

+ time_style = TextStyle()
+ time_style.font_size(36)
+ time_style.font("Arial")
+ time_style.align(TextStyle.RIGHT, TextStyle.TOP)

+ def annotation_from_label(frame):
+     developmental_hours = frame.volume.group_index * 0.5
+     frame.annotations.append(TextAnnotation(
+         f"{developmental_hours:.1f} hours", [0.99, 0.98], time_style))
+

+ for frame in frames:
+     annotation_from_label(frame)
```

```
...
```

Planar slicing of a static frame

To show the interior structure of a volume, one can interpolate a clipping plane across the volume. This can be done while the volume is changing over time, or with the volume held constant. Keyframes for the plane positions are loaded, then interpolated. Since the view is of a static volume, interpolation of the camera will not be necessary, however, the channel intensity compensation must still be applied. Here, we highlight in grey and use a red minus sign prefixing lines of code to indicate they have been deleted. An *AFrameSpan* containing only the last frame can be extracted by slicing relative to the end of the span via Python's negative indexing notation. One can multiply a frame span by an integer to produce a frame span containing that number of repetitions. Since the framerate was previously specified as 30 FPS, a multiple of that will yield the number of seconds to hold the volume static. The keyframes are interpolated over this static frame span to produce the planar clipping motion. The *end_continuity* parameter specifies the number of time-derivatives which should be zero at the ends of the interpolating spline. An end continuity of one indicates that the initial and final velocity of the clipping plane should be zero. Note that a relative time value of one does not need to be included in the interpolation, in which case, the remaining time in the span is extrapolated.

```
...
kf_chan = [
    AScene.load("kf_intensity0.json"),
    AScene.load("kf_intensity60.json"),
    AScene.load("kf_intensity120.json")
]
+ kf_cut_none = AScene.load("kf_plane_cut_none.json")
+ kf_cut_half = AScene.load("kf_plane_cut_half.json")
+ kf_cut_all = AScene.load("kf_plane_cut_all.json")

frames.apply_scene(scene_main)
- # One set of frames with evenly-spaced camera positions.
- frames.interpolate_camera_1d([0, 0.2, 0.4, 0.6, 0.8, 1], kf_cam)
frames.interpolate_channels_1d([0, 0.5, 1], kf_chan)
- anim.add_frames(frames)

+ # Show the clipping plane motion with a static volume for 9 sec at 30 FPS.
+ last_frame = frames[-1:]
+ static_frames = last_frame * (9 * 30)
+ static_frames.interpolate_planes_1d(
+     [0, 0.3, 0.6, 0.8],
+     [kf_cut_none, kf_cut_all, kf_cut_none, kf_cut_half],
+     end_continuity=1)
+ anim.add_frames(static_frames)
...
```

Rewinding and speed controls

The same set of frames can be added to the animation multiple times with changes in between additions, allowing for repeated visualizations or looping. Since this set of volumes does not naturally loop, one could simply add the frames again to replay it from the beginning, or they could play the frames in reverse. To make the reverse animation quick, a copy of the frames is made on a compressed time scale of 70 FPS for a 10x speed-up. These frames will need the annotations to be applied so that time can be shown running backwards.

```
...
frames = anim.make_frames(volume_rate=7, absolute_rate=True)
+ frames_fast = anim.make_frames(volume_rate=70, absolute_rate=True)
...
for frame in frames:
    annotation_from_label(frame)
+ for frame in frames_fast:
+     annotation_from_label(frame)
```

This animation combines the previous two animations, one after the other, with reversed playback in between. As such, the camera interpolation is re-added.

```
frames.apply_scene(scene_main)
+ # One set of frames with evenly-spaced camera positions.
+ frames.interpolate_camera_1d([0, 0.2, 0.4, 0.6, 0.8, 1], kf_cam)
frames.interpolate_channels_1d([0, 0.5, 1], kf_chan)
+ anim.add_frames(frames)
```

A “pause” is added at the end of the camera interpolation by selecting the last frame and adding it multiple times. With 30 frames added at 30 FPS, one second of pause results.

```
+ # Add some freeze-frames so we can visually isolate the rewind.
+ last_frame = frames[-1]
+ for i in range(30):
+     anim.add_frames(last_frame)
```

The fast frames exist independently of the regular frames, so the main scene and channel corrections must be reapplied. The reversal is done by slicing *frames_fast* with a step length of -1 to get a reversed sequence. Since the animation ends with the first frame, that frame is repeated to pause after rewinding.

```
+ # Play the fast frames backwards to get a "rewind" effect.
+ frames_fast.apply_scene(scene_main)
+ frames_fast.interpolate_channels_1d([0, 0.5, 1], kf_chan)
+ anim.add_frames(frames_fast[::-1])
+ # Freeze at the end of the rewind, too.
+ first_fast_frame = frames_fast[0]
+ for i in range(30):
+     anim.add_frames(first_fast_frame)
```

Instead of holding a static frame while the clipping plane passes through, the regular frames are used. Edited lines are shown with green greater-than signs.

```
- # Show the clipping plane motion with a static volume for 9 sec at 30 FPS.
- last_frame = frames[-1:]
- static_frames = last_frame * (9 * 30)
+ frames.apply_scene(scene_main)
+ frames.interpolate_channels_1d([0, 0.5, 1], kf_chan)
> frames.interpolate_planes_1d(
>     [0, 0.3, 0.6, 0.8],
>     [kf_cut_none, kf_cut_all, kf_cut_none, kf_cut_half],
>     end_continuity=1)
> anim.add_frames(frames)
...
```

Putting it all together

Here, the full animation script for a non-trivial visualization is shown. This animation incorporates all the elements previously discussed, starting with the camera interpolation, then pausing, rewinding, repeating with a static camera view, pausing again, interpolating a clipping plane over a static volume, rewinding while holding the clipping plane constant, and repeating once again with a cut-away view—while showing the timestamp annotation and correcting the channel intensity for all frames.

Notably unique in this script, the clipping plane is set to crop in the main scene by indexing the first (out of a list of one item) plane in the scene and setting “checked” to *True*.

```
import sys
sys.path.append("C:/.../Open_Chrono_Morph_Viewer")

from animation.animator import Animator
from animation.ascene import AScene
from animation.annotation import (
    TextAnnotation,
    TextStyle
)


title = "light_sheet_demo"
volume_files = "C:/.../DS1_4D/"
out_dir_path = f"C/.../videos/{title}/"
frame_size = (1920, 1080)  # Pixels (wide, high).

anim = Animator(30, out_dir_path, preview=False)
anim.set_volume_dir(volume_files)

frames = anim.make_frames(volume_rate=7, absolute_rate=True)
frames_fast = anim.make_frames(volume_rate=70, absolute_rate=True)

time_style = TextStyle()
time_style.font_size(36)
time_style.font("Arial")
```

```python
time_style.align(TextStyle.RIGHT, TextStyle.TOP)

def annotation_from_label(frame):
    developmental_hours = frame.volume.group_index * 0.5
    frame.annotations.append(TextAnnotation(
        f"{developmental_hours:.1f} hours", [0.99, 0.98], time_style))

for frame in frames:
    annotation_from_label(frame)
for frame in frames_fast:
    annotation_from_label(frame)

scale = frames.get_optimal_scale(frame_size[1])
scene_main = AScene.load("scene.json")
scene_main.items["Camera"]["scale"] = scale
kf_cam = [
    AScene.load("kf_cam0.json"),
    AScene.load("kf_cam1.json"),
    AScene.load("kf_cam2.json"),
    AScene.load("kf_cam3.json"),
    AScene.load("kf_cam4.json"),
    scene_main
]
kf_chan = [
    AScene.load("kf_intensity0.json"),
    AScene.load("kf_intensity60.json"),
    AScene.load("kf_intensity120.json")
]
kf_cut_none = AScene.load("kf_plane_cut_none.json")
kf_cut_half = AScene.load("kf_plane_cut_half.json")
kf_cut_all = AScene.load("kf_plane_cut_all.json")

# We always want the channels to be interpolated and default to the main
scene.
frames.apply_scene(scene_main)
frames.interpolate_channels_1d([0, 0.5, 1], kf_chan)
# One frame set with evenly spaced camera positions.
frames.interpolate_camera_1d([0, 0.2, 0.4, 0.6, 0.8, 1], kf_cam)
anim.add_frames(frames)

# Add some freeze-frames so we can visually isolate the rewind.
# Note: we are selecting a single `AFrame`. You can't interpolate this.
last_frame = frames[-1]
for i in range(30):
    anim.add_frames(last_frame)

frames_fast.apply_scene(scene_main)
frames_fast.interpolate_channels_1d([0, 0.5, 1], kf_chan)
# Play the fast frames backwards to get a "rewind" effect.
anim.add_frames(frames_fast[::-1])

# Freeze at the end of the rewind, too.
first_fast_frame = frames_fast[0]
for i in range(30):
    anim.add_frames(first_fast_frame)

# Go forward one more time without changing the camera position.
```

```
frames.apply_scene(scene_main)
frames.interpolate_channels_1d([0, 0.5, 1], kf_chan)
anim.add_frames(frames)

# Pause at the end for a second.
# Note: we are slicing to get an `AFrameSpan`. You can multiply this to
# create more frames.
last_frame = frames[-1:]
for i in range(30):
    anim.add_frames(last_frame)

# Show the clipping plane motion with a static volume for 9 sec at 30 FPS.
static_frames = last_frame * (9 * 30)
static_frames.interpolate_planes_1d(
    [0, 0.3, 0.6, 0.8],
    [kf_cut_none, kf_cut_all, kf_cut_none, kf_cut_half],
    end_continuity=1)
anim.add_frames(static_frames)

# Rewind again with the clipping plane active.
for frame in frames_fast:
    frame.scene.clipping_planes[0]["checked"] = True
anim.add_frames(frames_fast[::-1])

# Freeze for a second.
first_fast_frame = frames_fast[0]
for i in range(30):
    anim.add_frames(first_fast_frame)

# Play forward with the clipping plane active.
scene_main.clipping_planes[0]["checked"] = True
frames.apply_scene(scene_main)
frames.interpolate_channels_1d([0, 0.5, 1], kf_chan)
anim.add_frames(frames)

# Finally, freeze one last time.
last_frame = frames[-1]
for i in range(30):
    anim.add_frames(last_frame)

anim.render_frames(frame_size, keep_order=False)
anim.compile_video(out_dir_path + f"../{title}.mp4")
```

Supplementary Note 9: Comparison with existing volumetric time series software

Here, the authors detail their findings comparing the features provided by several common software packages which advertise their ability to process volumetric time series, also known as “4D images”, or sometimes labeled “5D” when multiple channels are also considered. These findings are summarized in **Supplementary Table 3**.

OCMV (this software package)

OCMV was originally conceived as an open-source substitute for the Imaris, a commercial volumetric time series visualization software. OCMV is intended to be used in conjunction with an analytical program such as ImageJ. As such, it does not reimplement the analytical functions provided by such software. This aspect is represented in row 11 of **Supplementary Table 3**. Note how comparable software provides various annotations, analytical functions, segmentation tools, cell trackers, tracers, plugins, or other tools for analyzing the data. In contrast, OCMV provides functions these otherwise comparable software lack, namely with its superior real-time volume clipping functions, including a clipping spline, live management of large numbers of files across two timelines, and support for sophisticated animation. All these functions are bundled into a straightforward user interface for use alongside other software. To support this simultaneous use, OCMV primarily supports the NRRD file format which is natively readable by most other applications. Volumes are stored as separate files to keep the individual file sizes small and transportable while avoiding single-use data structures such as Imaris’ file format.

The ideal use-case for this software consists of real-time inspection or scripted ray cast rendering of many single or multichannel time points with moderately sized individual volumes (<1 gigapixel each). The advanced clipping functions are rare among free software packages and are ideal for dealing with opaque or noisy data which cannot be readily thresholded for transparency.

Imaris

Imaris is powerful, but it is commercial software. Like OCMV, it supports multiple clipping planes, although it lacks a clipping spline. While it handles its timelines well and boasts an integrated animation rendering UI, the control of the transfer function leaves much to be desired, and the requirement of converting files to Imaris’ file format represents a major bottleneck in the data processing pipeline since all data must be contained in a single file, and the file processing time increased super linearly with the amount of data being processed.

The author’s experience with the software’s stability was lackluster, with many bugs apparent, and frequent fatal errors which required the author to restart their investigations from scratch. Even so, Imaris boasts powerful tools for particle tracking and data processing, with MATLAB integration and many segmentation functions equivalent to those in other packages.

Imaris represents a complete solution for data visualization and processing, although the price tag and lack of stability may prove prohibitive.

napari

The basic form of napari is simple and comparatively limited; this software is most useful in conjunction with custom Python scripts to collect data files and specify the visualization setup. napari provides the most freedom of these software in its rendering and visualization capabilities, but it does not wrap that functionality in a graphical user interface, and as such, although it includes a stand-alone component, it should be thought of as more of a Python library like matplotlib than as a fully-fledged application. This software is unique in that it allows one to combine multiple overlapping types of data, such as voxel images and surface mesh data into a composite scene with relative ease, along with support for an unlimited number of non-spatial dimensions on top of the 3D view by simply adding additional sliders.

The napari viewer represents an advanced tool for users who are comfortable writing Python and able to leverage the vast collection of libraries available.

Icy

Of the software considered, Icy is the closest equivalent free software to OCMV. It has excellent support for previewing image files with its Bioimage Formats integration and for combining separate volumes into a single image in an intelligent way based on the file names. Like OCMV, it uses a VTK backend for rendering volume data and has a clipping plane, although only one plane is currently supported in the 3D viewer. It has excellent support for ROIs and annotation, along with a clean and attractive user interface. It deals well with large numbers of files that are open simultaneously, and it has rudimentary cache management for virtual images larger than the available RAM. Uniquely, it bundles a copy of ImageJ which can be used to pass images between the two software packages without writing them to a file in between, although a non-trivial delay is incurred when transferring the image between the two workflows.

Icy is most useful for users looking to use its high-quality 3D annotation tools in conjunction with the data processing plugins from ImageJ.

ImageJ

ImageJ is a long-standing tool specializing in 2D image analysis but remaining useful for 3D and 4D data with its hyperstack feature. Written in Java, it specializes in its broad array of plugins with support for multiple scripting languages, including ImageJ macro. It can import and export practically any image file format in common use, leading it to act as the common interface between the various methodologies of the bioimaging community. Its 3D rendering capabilities are limited, however, and it only supports certain types of files as “virtual images” which may be larger than RAM. ImageJ lacks common 3D visualization tools such as ortho slicers to view multiple image planes simultaneously, and while it technically supports 3D time series playback via a plugin, its capabilities are cumbersome and lackluster compared to the modern alternatives.

ImageJ is most useful when it comes to converting between file formats, quickly inspecting images from a limited 2D perspective, making simple measurements, and leveraging its deep reservoir of image processing plugins.

Vaa3D

Like ImageJ, Vaa3D has particularly nice tools for 2D image processing, and especially neuron tracing since it was developed for mapping the brains of fruit flies. However, it improves upon the 3D visualization capabilities with simple 3D renderings and projections. It has rudimentary support for animations to rotate the volume in place, although its support for temporal data and visualization capabilities are relatively weak. 3D viewing of large datasets must be done via the TeraFly module, which requires image data to be converted to a specific directory structure. The image file formats it can handle are limited, including TIFF images, NRRD, and their own Vaa3Draw format.

Vaa3D is most useful for those performing image operations such as neuron tracing or segmentation, where 3D renderings are helpful, but not essential.
